# A French-Language Web-Based Intervention Targeting Prolonged Grief Symptoms in People Who Are Bereaved and Separated: Randomized Controlled Trial

**DOI:** 10.2196/57294

**Published:** 2024-10-16

**Authors:** Anik Debrot, Liliane Efinger, Maya Kheyar, Valentino Pomini, Laurent Berthoud

**Affiliations:** 1 Institute of Psychology Faculty of Social and Political Sciences University of Lausanne Lausanne Switzerland

**Keywords:** web-based interventions, randomized controlled trial, grief, bereavement, separation, guidance

## Abstract

**Background:**

Losing a loved one, through death or separation, counts among the most stressful life events and is detrimental to health and well-being. About 15% of people show clinically significant difficulties coping with such an event. Web-based interventions (WBIs) are effective for a variety of mental health disorders, including prolonged grief. However, no validated WBI is available in French for treating prolonged grief symptoms.

**Objective:**

This study aimed to compare the efficacy and adherence rates of 2 WBIs for prolonged grief symptoms following the loss of a loved one through death or romantic separation.

**Methods:**

LIVIA 2.0 was developed relying on theoretical and empirical findings on bereavement processes and WBIs, and is compared with LIVIA 1, which has already demonstrated its efficacy. We conducted a randomized controlled trial and provided on-demand guidance to participants. Outcomes were assessed through web-based questionnaires before the intervention, after the intervention (12 weeks later), and at follow-up (24 weeks later). Primary outcomes were grief symptoms, depressive symptoms, and well-being. Secondary outcomes were anxiety symptoms, grief coping strategies, aspects related to self-identity, and program satisfaction.

**Results:**

In total, 62 participants were randomized (intent-to-treat [ITT] sample), 29 (47%) in LIVIA 2.0 (active arm) and 33 (53%) in LIVIA 1 (control arm). The dropout rate was 40% (37/62), and 10 participants were removed due to exclusion criteria, leading to a final per-protocol sample of 27 (44%) completers who differed from noncompleters only based on reporting fewer anxiety symptoms (t_60_=3.03; *P*=.004). Participants who are separated reported more grief symptoms (t_60_=2.22; *P*=.03) and attachment anxiety (t_60_=2.26; *P*=.03), compared to participants who are bereaved. There were pre-post within-group differences for both programs in the ITT sample, with significant reductions in grief (Cohen *d*=−0.90), depressive symptoms (Cohen *d*=−0.31), and centrality of the loss (Cohen *d*=−0.45). The same pattern was observed in the per-protocol sample, with the exception that anxiety symptoms also significantly diminished (Cohen *d*=−0.45). No difference was found in efficacy between the 2 programs (all *P*>.33). Participants (ITT sample) reported overall high levels of program satisfaction (mean 3.18, SD 0.54; over a maximum of 4). Effect stability was confirmed at the 6-month follow-up for all outcomes, with an improvement in self-concept clarity.

**Conclusions:**

The 2 grief-related WBIs were effective in reducing grief, depressive and anxiety symptoms for participants who are bereaved or separated. The analyses did not reveal any pre-post between-group differences, suggesting that the innovations brought to LIVIA 2.0 did not significantly affect the outcome. However, caution is warranted with the interpretation of the results given the limited power of the sample, which only allows the detection of medium effect sizes.

**Trial Registration:**

ClinicalTrials.gov NCT05219760; https://clinicaltrials.gov/study/NCT05219760

**International Registered Report Identifier (IRRID):**

RR2-10.2196/39026

## Introduction

### Background

Distress following the loss of a loved one is a painful yet normal reaction. While most individuals recover over time, some experience prolonged grief symptoms, characterized by intense feelings of grief that persist for an extended period [1,2]. Face-to-face interventions show moderate to large effect sizes to treat these symptoms [3,4] but lack accessibility (eg, [5,6]). Web-based interventions (WBIs) can help improve accessibility and provide numerous efficient prevention and treatment programs for a variety of psychological difficulties [7,8]. Notably, WBIs have demonstrated effectiveness in addressing prolonged grief symptoms, yielding moderate to large effect sizes [9,10]. These interventions are generally based on methods derived from empirically supported face-to-face psychological interventions.

A common means to enhance the effectiveness of WBIs [11], including those targeting prolonged grief symptoms [9], is to provide guidance to participants (ie, “any direct and bidirectional communication with the individual designed to support the clinical aspects of the intervention, facilitate intervention completion, and/or achieve the desired clinical outcomes” [11]). However, recent evidence suggests that the impact of guidance on effect sizes is lower in more interactive internet interventions (ie relying on technologies such as videos, audios or gamification features) [12]. In addition, when given the option, not all participants request guidance, yet the efficacy of guidance on demand condition is similar to that of standard weekly guidance [13,14].

LIVIA 1 is a WBI program designed to treat prolonged grief symptoms following bereavement or separation [15]. Fundamental research indicates that both types of losses involve very similar underlying processes (eg, [16,17]). LIVIA 1 was assessed in German through a randomized controlled trial [18] and in French through a noncontrolled trial [19]. These studies demonstrated that the same intervention can be efficiently administered to both populations. A detailed description of the LIVIA 1 intervention is available in the protocol by Brodbeck et al [15].

For this study, we developed LIVIA 2.0, an upgraded version of LIVIA 1. This program integrates recent developments in WBIs [20,21] and incorporates various elements to enhance patient adherence and program efficacy while reducing the need for guidance. Specifically, a series of changes were designed to improve participant autonomy. First, we sent automated emails [22] in two situations: (1) to announce to the participants that a new session is available and (2) in case the participant has not accessed the intervention for 7 consecutive days. Second, we more closely tailored the intervention to each participant in two ways: (1) by providing automated individualized recommendations about the module completion order [23] and (2) by proposing at each session a choice of different exercises that meet different situations or needs. More specifically, we evaluated each participant’s priorities and recommended the order of the modules accordingly in the first session. In each session, we provided 3 choices of exercises so that the participant could choose what suited their needs best. Third, we evaluated, promoted, and encouraged the use of personal resources based on a validated self-assessment tool, the resources self-assessment scale [21]. Finally, relying on research showing the benefits of augmented interactivity [20,24,25], we developed more interactive content in the form of psychoeducation videos and quizzes. Apart from the introductory and concluding sessions, the structure of LIVIA 2.0 revolves around 4 modules focusing on key cognitive behavioral therapy topics: thoughts, behaviors, and emotions. Moreover, we developed a module based on empirical cognitive psychopathological knowledge that addresses identity and memory processes, which are crucial for adapting to loss [26,27]. Autobiographical memory refers to memories from past personal experiences. It serves to maintain self-continuity and provides the ability to stay oriented in the world and pursue goals [28,29]. In the grief context, the loss of a significant other is often a life-changing event that can disrupt one’s life story, sense of self, and future plans [27]. Therefore, addressing these disturbances can play a crucial role in alleviating prolonged grief symptoms by helping individuals develop a more adaptive and coherent sense of self. Given these considerations, we aimed to include measures of three key identity-related variables in our study: (1) self-continuity, which refers to the perception of a coherent connection between one’s past, present, and future self [30]; (2) self-concept clarity, which refers to the clear and coherent understanding of one’s own traits, beliefs, and values [31]; and (3) event centrality, which refers to the extent to which individuals construct the traumatic event as a reference point to understand themselves and the world [32]. By doing so, we aim to provide a more comprehensive understanding of how the LIVIA interventions impact these facets of identity. An overview of the content of LIVIA 2.0 can be found in Table 1.

**Table 1:** Overview of the sessions and key content of the LIVIA 2.0 intervention (active arm) targeting prolonged grief in individuals who are bereaved or separated.

Session	Module^a^	Theme	Content
1	Introduction	Psychoeducation plus resources and goals assessment	Information about the self-help intervention, grief reactions, predictors, and treatment of prolonged grief. Assessment of personal resources and goals in pursuing the intervention
2	Cognition-focused	Loss-oriented session^b^	Information about the impact of negative thoughts on well-being and the typical negative thoughts experienced during difficult grief. Cognitive restructuration exercises.
3	Cognition-focused	Restoration-oriented session^c^	Information about secondary stressors and related thoughts. Importance of building positive thoughts as resources. Exercise to promote focus on positive aspects of one’s own life.
4	Emotion-focused	Loss-oriented session	Information about the central role of emotions in the grieving process. Assessment of own emotional state. Auto-compassion exercises.
5	Emotion-focused	Restoration-oriented session	Importance of experiencing positive emotions, even if only briefly. Hypnosis-like exercises to promote positive emotions.
6	Behavior-focused	Loss-oriented session	Information about the typical vicious circle of avoidance in grief and the importance of confrontation to the avoided situations. Confrontation exercises.
7	Behavior-focused	Restoration-oriented session	Importance of behavioral activation in line with one’s own values. Assessment of values. Preparation of behavioral activation in line with one’s own values.
8	Identity-focused	Loss-oriented session	Psychoeducation about identity formation and the way it is affected by grief. Exercise: revisiting memories and the relationship with the lost person with an independent sense of identity.
9	Identity-focused	Restoration-oriented session	Psychoeducation about the importance of autobiographical memory for the individual’s sense of self and ability to generate images of future events. Exercise aimed at focusing on specific adaptive autobiographical memories and future projections to foster an independent self-identity.
10	Conclusion	Assessment of the experience of the intervention+ relapse prevention	Promoting reflection on one’s own journey through the program (what was learned and what still needs to be done)+identification of vulnerable moments and strategies to deal with the latter.

^a^Modules 2 to 9 can be completed in any order selected by the participants, based on the personalized recommendations provided by the program at the end of session 1.

^b^Loss-oriented refers to focusing on thoughts and feelings related to the loss.

^c^Restoration-oriented refers to focusing on life changes and new roles or responsibilities following the loss.

The innovations in LIVIA 2.0 were also developed based on the theoretical and empirical literature on grief and romantic dissolution. Theoretically, we relied on one of the most influential models of coping with loss, the dual process model (DPM) of coping with bereavement [33,34]. According to this model, instead of progressing through consecutive phases, individuals oscillate between focusing on loss-oriented thoughts and feelings and focusing on restoration from the loss (ie, life changes and new roles or responsibilities following the loss). This oscillation is considered a natural and necessary process for coping with loss. In addition, evidence suggests that DPM-based interventions may be more effective than traditional ones [35]. LIVIA 2.0 was designed to mimic the oscillation process by alternating between loss- and restoration-focused sessions within each of its 4 modules. Furthermore, LIVIA 2.0 incorporates recent empirical findings related to loss into its content and exercises, such as self-compassion exercises, which predict better grief recovery [36,37]. Exploratory analyses of the use and potential impact of the innovations included in LIVIA 2.0 were conducted, particularly in relation to guidance requirements, automated emails, reliance on personal resources, and the identity module [38]. Given the combination of empirically-based changes implemented in LIVIA 2.0, we expect it to be more efficient than LIVIA 1 when provided in a guidance on demand format.

### Objectives

Our main hypotheses are as follows: (1) both LIVIA 1 and LIVIA 2.0 will increase participants’ well-being and decrease their mental health symptoms after the intervention and at follow-up, (2) LIVIA 2.0 will be more efficient than LIVIA 1 across all outcomes, and (3) LIVIA 2.0 will have a lower dropout rate than LIVIA 1. In addition, we will compare participant satisfaction between both versions.

These hypotheses were preregistered in a published protocol [39], although not all are addressed in this study. First, the comparison of guidance requirements between LIVIA 2.0 and LIVIA 1, as well as part of the qualitative investigation of the semantic content of the responses to the LIVIA 2.0 exercises are discussed in other publications [38,40]. Second, the smaller sample size obtained, compared to the target, neither provides sufficient statistical power to analyze the short-term effectiveness of each LIVIA 2.0 module on participants’ weekly moods, feelings of loneliness, and prolonged grief symptoms, nor to explore the role of multiple measures as moderators of the program’s efficacy.

## Methods

This study is a monocentric, single-blinded, 2-arm randomized controlled trial comparing the efficacy of 2 versions of a French-language WBI, LIVIA 1 and LIVIA 2.0, designed to alleviate mental health symptoms and enhance the well-being of individuals experiencing prolonged grief symptoms following the loss of a loved one.

### Study Conditions

In both study conditions, participants received automated emails if they had not accessed the intervention platform for a week. In addition, they could request guidance whenever needed.

LIVIA 1 is a 10-session self-help intervention designed to address prolonged grief symptoms resulting from the death of, or separation or divorce from, a romantic partner, as developed by Brodbeck et al [15]. Participants are encouraged to complete 1 session per week, with each session estimated to take about 1 hour, working through exercises provided in downloadable PDF files. Each session includes various texts, audio files, exercises, and interactive quizzes, and must be completed in the prescribed order [39]. This intervention serves as the control condition and its efficacy has been previously demonstrated [18].

LIVIA 2.0 is a psychological WBI developed by the authors of this study, consisting of 10 sessions [39,41]. Each session takes approximately 30 to 45 minutes to complete and includes an introductory session, 8 sessions divided into 4 modules, and a concluding session. The modules cover 4 main themes: cognitions, emotions, behaviors, and identity. On the basis of the results of a short questionnaire, an individual recommendation for the order of module completion is provided at the end of the introductory session. Theoretically anchored in the DPM [34], each module comprises of first session focused on loss and a second on restoration. Each session features psychoeducational information and 3 versions of an exercise related to the session’s main theme. Participants are expected to complete at least 1 exercise per session, choosing the one that best suits them, though they can complete all the exercises if they wish. LIVIA 2.0 incorporates various exercises, texts, audio and video files, and interactive quizzes. Participants in this condition can access a maximum of 1 session per week and receive an automated email when a new session becomes available. This set-up serves as the active condition in this study. Previous versions of the modules were qualitatively pretested on small samples as part of 6 masters’ theses [42-47], and the intervention was adapted based on the results. The content of the intervention was frozen during this trial. Both interventions were hosted on a website developed by RationalK SàRL.

### Recruitment

Participants were recruited from French-speaking regions of Switzerland through various methods. Recruitment was conducted by contacting associations (eg, grief- and divorce-related organizations, older adult groups, and neighborhood associations), engaging with media outlets (radio, television, and newspapers), distributing flyers in public locations (eg, beauty salons and churches), emailing university student groups, promoting the study through social media (Facebook [Meta Platforms, Inc] and Instagram [Meta Platforms, Inc]), and posting advertisements on research facility websites. Recruitment lasted from May 2022 to January 2023, with the last participant completing the follow-up in August 2023. We concluded recruitment due to time and funding constraints. Our institutional affiliation was displayed on all recruitment material, including posters, website, social media posts, and flyers and was mentioned in all media appearances, such as radio interviews and press articles. All participants were required to fill out an informed consent form, which they downloaded on the internet along with an information sheet. We provided our contact information in multiple locations to ensure participants could easily reach out with any questions.

### Ethical Considerations

The research protocol was approved by a federally-recognized state ethics committee (Commission cantonale d’éthique de la recherche sur l’être humain BASEC 2021-D0086) and the Swiss Agency for Therapeutic Products (Swissmedic; 102667545) in accordance with Swiss Ordinance 810.306 on Clinical Trials with Medical Devices. The trial was registered on ClinicalTrials.gov (NCT05219760).

All participants were required to complete an informed consent form, which was made available for downloading on the web along with an information sheet. The content of these documents is available in Multimedia Appendix 1. Contact information was provided in multiple locations for participants to reach out to the research team with any queries. In accordance with Swiss legislation and ethical standards, participants were required to provide their signature on the informed consent form. Subsequently, participants were given the option to either scan and email the signed informed consent form or to send it by post.

To guarantee the highest level of participant safety, the suicidal risk of interested individuals was initially evaluated using the 5-item Suicidal Ideation Attributes Scale (SIDAS) [48]. Those who met the validated risk threshold (≥20 [49]) were excluded from participation and provided with information regarding the availability of appropriate support. Individuals with a low risk (SIDAS score 0-12) were automatically admitted for participation. For individuals with a medium risk (SIDAS score 13-19), a phone-based clinical interview was conducted to ensure an optimal assessment of suicidal risk and referral for appropriate treatment using the Risk-Urgency-Danger procedure [50]. Furthermore, the assessment of suicidal risk and the aggravation of symptomatology (≥1 SD for grief and depressive symptoms) was conducted at the posttest and follow-up stages.

Personal (identifiable) data (name, phone number, email address, and birthdate) were asked on the informed consent form. This information was collected via email or mail and stored on a network-attached storage system provided by the University of Lausanne. All other data were encrypted and stored on secure servers. Participants were not offered any form of compensation.

### Participants and Procedure

#### Inclusion and Exclusion Criteria

Inclusion criteria were: (1) having experienced bereavement or separation more than 6 months before participation, (2) feeling the need for support to cope with the loss, (3) being >18 years of age, (4) having regular access to the internet and basic computer and internet literacy, (5) speaking French fluently, and (6) having provided written approval of the informed consent form.

Exclusion criteria were: (1) the presence of moderate to acute suicidality (assessed before the start of the program), (2) the presence of severe psychological or somatic disorders requiring immediate treatment, (3) concomitant psychotherapy, (4) the prescription or dosage change of psychoactive drugs in the month before or during the program, (5) the inability to follow the study procedures, and (6) enrollment of the investigators, their family members, employees, and other dependent people.

#### Sample Size and Condition Assignment

Among the 232 individuals who clicked on the screening questionnaire while visiting the program website, 137 (59.1%) were accepted into the study. In total, 73 (53.3%) of the 137 individuals did not send back the informed consent form, and 2 (1.5%) did not complete the preintervention questionnaires, resulting in a total of 62 (45.3%) participants starting the program. These participants were included in the intent-to-treat (ITT) analyses. Out of these 62 participants, 33 (53%) participants were randomized into the LIVIA 1 condition and 29 (47%) participants into the LIVIA 2.0 condition. We used the randomization module in REDCap (Research Electronic Data Capture; Vanderbilt University) [51,52], which generates randomization automatically. We applied a single-blinded randomization strategy, stratified according to gender and loss type (bereavement vs separation), with randomization blocks of 10 persons, with an allocation of 1:1. Due to dropouts and exclusions for not meeting inclusion criteria (starting another treatment during the program; LIVIA 2.0: 3/62, 5% and LIVIA 1: 2/62, 3% and not completing at least 1 full session; LIVIA 2.0: 3/62, 5% and LIVIA 1: 2/62, 3%), 27 (44%) of the 62 participants were finally included into the per-protocol analyses (LIVIA 1: 15/27, 56% and LIVIA 2.0: 12/27, 44%). Measurements were taken at 3 points: before intervention, after intervention (12 weeks after the beginning of the intervention), and at follow-up (12 weeks after the end of the intervention). Participants who were randomized received a link to create an account on the intervention platform corresponding to their assigned intervention. They were then free to access the intervention at the pace they wished, although a weekly session was recommended.

### Primary Outcome Measures

All outcomes were assessed via self-report questionnaires that were completed on the web by participants on the REDCap platform [51,52] of the Centre Hospitalier Universitaire Vaudois, the Lausanne University Hospital. Participants were invited to complete the different questionnaires through an email containing a personalized link. If the questionnaires were not completed within a week, up to 3 reminder emails were sent at each stage (preintervention, postintervention, and at follow-up).

Prolonged grief symptoms were assessed with the Traumatic Grief Inventory Self-Report [53] (French translation by Cherblanc, J, unpublished data, September 2021). This 18-item self-report measure assesses the presence of symptoms on a 5-point scale, ranging from 1 (never) to 5 (always) [53]. This inventory is designed to evaluate symptoms of persistent complex bereavement disorder, as defined in the *Diagnostic and Statistical Manual of Mental Disorders*, *Fifth Edition* [1], and prolonged grief disorder, as per the *International Classification of Diseases, 11th Edition* [54]. It demonstrates good reliability and validity in identifying individuals at risk for prolonged grief disorder.

Depression symptoms were assessed with the Patient Health Questionnaire–9 [55], a 9-item measure of depression with adequate reliability and validity [56]. This questionnaire assesses various depressive symptoms over the previous 2 weeks on a scale ranging from 0 (never) to 3 (almost every day).

Well-being was measured with the French version [57] of the Flourishing Scale [58], a brief 8-item instrument of self-perceived success in important life areas such as relationships, self-esteem, purpose, and optimism. This scale assesses eudemonic well-being, a broader conception of conventional well-being measures. Participants responded to items such as “I lead a purposeful and meaningful life” on a scale ranging from 1 (strongly disagree) to 7 (strongly agree).

### Secondary Outcome Measures

Anxiety symptoms were measured with the Generalized Anxiety Scale [59,60], which includes 7 items (eg, “feeling nervous, anxious, or on edge”). Participants rated the frequency of symptoms over the previous 2 weeks on a 4-point Likert scale (0=not at all to 3=nearly every day).

Feelings of loneliness were assessed with the University of California Los Angeles Loneliness Scale [61,62] which contains 10 positive items (eg, “I feel in tune with the people around me”) and 10 negative items (eg, “I lack companionship”). Participants responded on a 4-point scale (1=never to 4=often).

Identity-related concepts were evaluated with 3 different scales. First, the 12-item Self-Concept Clarity Scale in its French version [31,63] assesses the clarity, consistency, and stability of self-beliefs. Participants answered on a 5-point scale ranging from 1 (strongly disagree) to 5 (strongly agree). Second, the Centrality of Event Scale [32] (French version by Ceschi et al [64]), assesses the extent to which a distressing life event serves as a reference point for personal identity and meaning attribution to other personal experiences. Responses were rated on a 5-point scale (1=totally disagree to 5=totally agree). Finally, 3 items assessed self-continuity [65]: “I am the same person as I always was,” “With time a lot of things have changed, but I’m still the same person,” and “I am a different person than I was in the past.” These items were evaluated on a 5-point scale (ranging from 1=does not apply to me at all to 5=fully applies to me).

Finally, satisfaction with the program was measured with a translated and adapted version of the Client Satisfaction Questionnaire adapted to internet-based interventions [66]. We included open-ended questions to obtain qualitative feedback on the intervention.

### Statistical Analysis

All analyses were conducted using SPSS (version 25; IBM Corp). We performed both ITT and per-protocol analyses. The ITT analyses included all participants randomized into one of the experimental conditions (N=62), while the per-protocol analyses included only those participants who completed all protocol requirements (27/62, 44%; refer to the study by Gupta [67]). For the descriptive characteristics of the sample at baseline, we tested differences between both experimental arms using 2-tailed *t* tests for continuous variables and chi-square tests for categorical variables, based on the ITT sample. To test hypotheses (1) and (2) mentioned in the Objectives section, we used multilevel mixed-effects models for repeated measures to evaluate the efficacy of LIVIA 2.0 compared with LIVIA 1 and the stability of the effects. These models account for the dependency of the data and the correlation of repeated measures within individuals, using all available data from each participant and estimating parameters for missing values [68].

Due to difficulties in participant recruitment, our sample size was significantly smaller than targeted. Post hoc analysis revealed that the statistical power of the per-protocol sample was adequate (0.80) to compute within-between-person interactions for a medium effect size, but insufficient to detect small within-between effect sizes (0.18) or medium between-person differences (0.33). Consequently, we adapted the analyses and decided not to conduct most secondary analyses.

## Results

### Baseline Characteristics

Table 2 presents the sociodemographic characteristics of the randomized sample, consisting of 62 French-speaking adults. Table 3 details the characteristics of the loss and the personal state of the randomized participants. We compared participants who are separated and bereaved at baseline on the characteristics outlined in Tables 2 and 3. In total, 3 differences emerged: participants who are separated reported more grief symptoms (t_60_=2.22; *P*=.03), higher attachment anxiety (t_60_=2.26; *P*=.03), and were more frequently in a current romantic relationship (t_60_=4.75; *P*<.001) compared to participants who are bereaved. No other significant differences were found (*P*>.20). A Bonferroni correction yields an α=.004. Hence, only the relationship status was different between both groups.

**Table 2:** Sociodemographic characteristics of the intention-to-treat sample before intervention for LIVIA 2.0 (active arm) and LIVIA 1 (control arm).

		Total (N=62)	LIVIA 2.0 (n=29)	LIVIA 1 (n=33)	Difference between both intervention arms	
Age (y), mean (SD)		45.2 (13.8)	46.76 (14.28)	43.85 (13.49)	t_60_=–0.82; *P*=.41	
**Gender (n=62)**						*χ*^2^_1_=0.1; *P*=.77
	Men	48 (77)	23 (79)	25 (76)		
	Women	14 (23)	6 (21)	8 (24)		
**Mother tongue (n=62)**						*χ*^2^_1_=2.4; *P*=.20
	French	56 (90)	1 (3)	28 (85)		
	Other	6 (10)	28 (97)	5 (15)		
**Currently in a relationship (n=62)**						*χ*^*2*^_1_=0.1; *P*=.99
	Yes	31 (50)	14 (48)	17 (52)		
	No	31 (50)	15 (52)	16 (48)		
**Education level (n=62)**						*χ*^*2*^_1_=5.9; *P*=.33
	Compulsory school	1 (2)	0 (0)	1 (3)		
	Apprenticeship	12 (19)	5 (17)	7 (21)		
	High school	5 (8)	4 (14)	1 (3)		
	Technical college	4 (6)	1 (3)	3 (9)		
	Higher professional education	2 (3)	0 (0)	2 (6)		
	University	38 (61)	19 (66)	19 (58)		
**Professional status (n=62)**						*χ*^*2*^_4_=2.5; *P*=.65
	Unemployed	7 (11)	2 (7)	5 (15)		
	In training	6 (10)	3 (10)	3 (9)		
	Part time	26 (42)	13 (45)	13 (39)		
	Full time	19 (31)	8 (28)	11 (33)		
	Other	4 (6)	3 (10)	1 (3)		

**Table 3:** Characteristics of the loss and personal state of intent-to-treat sample before intervention.

			Total (N=62)		LIVIA 2.0^a^, (n=29)		LIVIA 1^a^, (n=33)		Difference between both intervention arms	
**Type of loss (n=62)**										*χ*^*2*^_1_=0.01; *P*=.99
	Death	36 (58)		17 (59)		19 (58)				
	Separation	26 (42)		12 (41)		14 (42)				
**Person lost (n=62)**										*χ*^*2*^_4_=4.7; *P*=.76
	Partner	27 (44)		11 (38)		16 (48)				
	Mother	13 (21)		5 (17)		8 (24)				
	Father	6 (10)		3 (10)		3 (9)				
	Brother	5 (8)		2 (7)		3 (9)				
	Child	2 (3)		1 (3)		1 (3)				
	Other family members	5 (8)		4 (14)		1 (3)				
	Friend	1 (2)		1 (3)		0 (0)				
	Others	3 (5)		2 (7)		1 (3)				
Time since loss (y), mean (SD)			3.2 (6.4)		4.28 (9.03)		2.27 (2.17)		t_30.93_=–1.17; *P=.*25	
Relationship length (y), mean (SD)			24.0 (17.4)		23.7 (18.0)		24.3 (17.2)		t_22_=–0.52; *P=*.61	
**Loss expected (n=62)**										*χ*^*2*^_4_*=*4.85; *P=*.32
	Not at all	24 (39)		11 (38)		13 (39)				
	A little	14 (23)		7 (24)		7 (21)				
	Moderately	8 (13)		6 (21)		2 (6)				
	A lot	7 (11)		3 (10)		4 (12)				
	Completely	9 (15)		2 (7)		7 (21)				
**Loss experienced (n=62)**										*χ*^*2*^_3_=0.97; *P*=.82
	Very negatively	33 (53)		15 (52)		18 (54)				
	Negatively	16 (26)		9 (31)		7 (21)				
	Neutral	8 (13)		3 (10)		5 (15)				
	Positively	5 (8)		2 (7)		3 (9)				
Grief^b^			55.79 (12.17)^c^		56.0 (12.1)		55.6 (12.4)		t_60_=–0.14; *P*=.89	
Depression^d^ (sum)^e^			8.55 (4.64)^f^		9.17 (5.27)		8.00 (4.02)		t_60_=–0.99; *P*=.32	
Anxiety^g^			1.21 (0.78)		1.31 (0.85)		1.13 (0.72)		t_60_=–0.88; *P*=.38	
Well-being^h^			4.97 (0.99)		4.82 (0.92)		5.10 (1.05)		t_60_=1.09; *P*=.28	
Loneliness^i^			2.17 (0.61)		2.18 (0.63)		2.16 (0.60)		t_60_=–0.132; *P*=.89	
**Identity scales^j^**										
	Self-Concept Clarity	3.16 (0.83)		3.27 (0.94)		3.06 (0.73)		t_60_=–0.98; *P*=.33		
	Centrality of Event	3.73 (0.95)		3.72 (0.82)		3.73 (1.06)		t_60_=0.02; *P*=.98		
	Self-Continuity	2.66 (0.88)		2.77 (0.76)		2.57 (0.97)		t_60_=–0.91; *P=*.36		

^a^LIVIA 1 is the control arm, and LIVIA 2.0 is the active arm.

^b^Grief symptoms were assessed using the Traumatic Grief Inventory.

^c^40% above clinical cut-off (≥59).

^d^Depression symptoms were assessed using the Patient Health Questionnaire.

^e^The score was computed by summing the score at each item.

^f^Minor: 24%, mild: 37%, moderate: 27%, moderately severe: 10%, and severe: 2%.

^g^Anxiety symptoms were assessed using the Generalized Anxiety Disorder-7.

^h^Well-being was assessed using the Flourishing Scale.

^i^Loneliness was assessed using the University of California, Los Angeles Loneliness Scale.

^j^Identity scales used were the Self-Concept Clarity Scale, Centrality of Event Scale, and Self-Continuity items.

### Adherence to Treatment and Dropout Analysis

**Figure 1:**
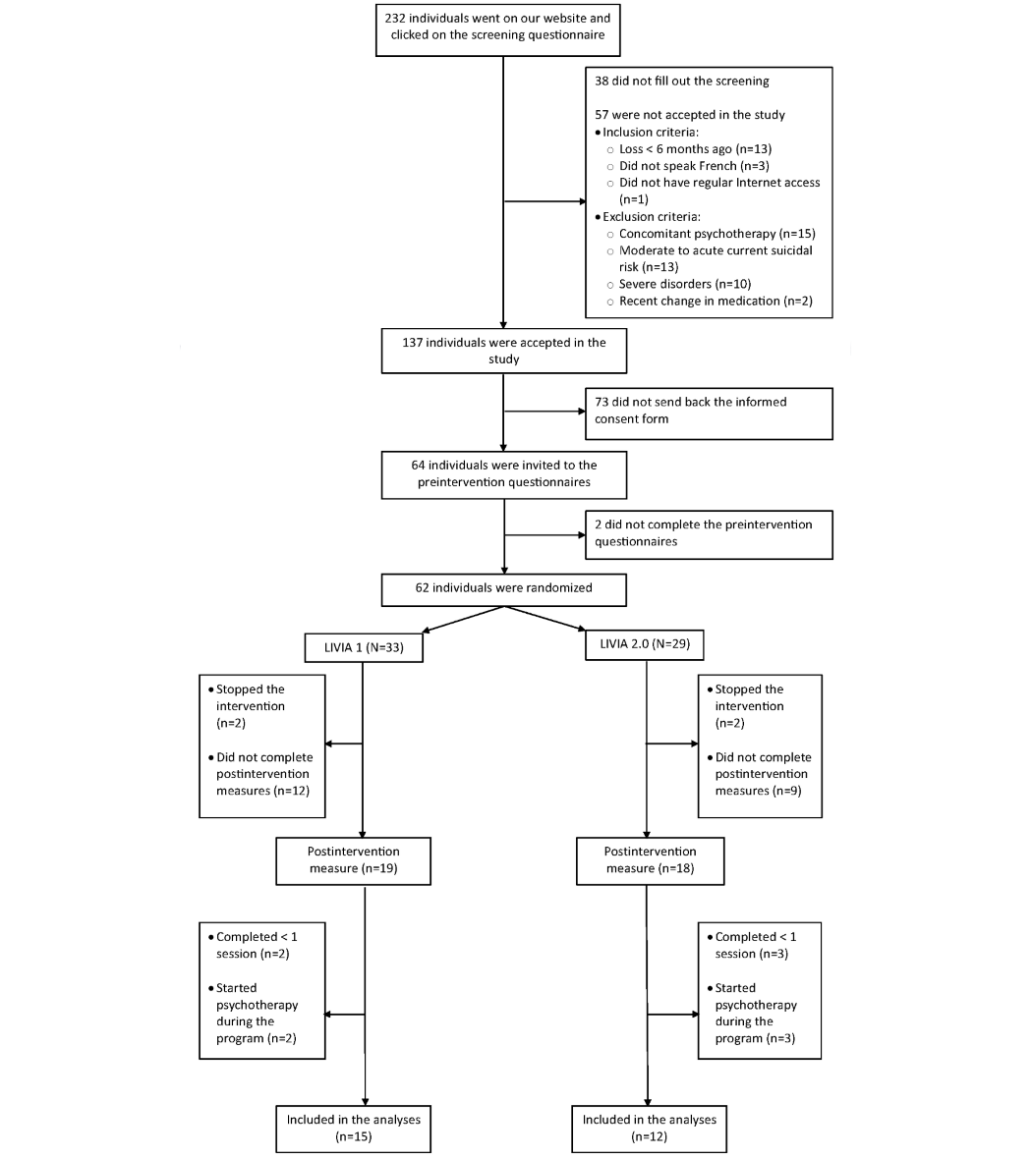
Study design flowchart. LIVIA 2.0: active arm and LIVIA 1: control arm.

Among the 62 randomized participants, 37 (60%) completed the postintervention measures. In addition, 5 (8%) of the 62 participants were excluded because they did not complete at least 1 entire program session, and another 5 (8%) were excluded for starting psychotherapy during the program. Consequently, the final sample of completers comprised 27 participants (44% of the randomized sample, no difference between both arms, t_60_=0.32; *P*=.75; Figure 1), which is considerably fewer than what was planned (234 participants at posttest stage). Completers and noncompleters only differed on anxiety symptoms, with completers having reported fewer symptoms compared to noncompleters (t_60_=3.03; *P*=.004).

### Overall Effects at Posttreatment Stage

Table 4 presents the results of the mixed-effects model analyses, and means and SDs for all outcomes for the ITT analyses. Several outcomes demonstrated pre-post within-group differences. Indeed, both LIVIA programs resulted in significant and large reductions in grief symptoms, medium reductions in depressive symptoms, and a medium effect size reduction in the perceived centrality of the loss at posttest stage. Anxiety symptoms showed a trend toward reduction, with a medium effect size, but without reaching significance (*P*=.07). However, well-being, loneliness, self-concept clarity, and sense of self-continuity did not significantly change over the treatment period. However, neither group-by-time interaction nor between-group effect was found for any outcomes, indicating that the pre-post within-group differences were not different between the 2 programs. The results can be found in Table S1 of Multimedia Appendix 2. Given the small sample size and low statistical power, it is likely that some small effects were undetected. The per-protocol analyses results, detailed in Table S2 of Multimedia Appendix 2, showed a similar pattern except for anxiety symptoms, which only showed a fully significant reduction within group.

**Table 4:** Within-person effects of LIVIA 2.0 (active arm) and LIVIA 1 (control arm) in the intention-to-treat sample (N=62).

Domain and arm^a^		Preintervention			Postintervention			Pre-post within-group^b^
		Scores, mean (SD)	Participants, n^c^	Scores, mean (SD)		Participants, n (%)		
**Grief^d^**							β=–.64; t_44.07_=–4.94; *P*<.001; 95% CI –0.90 to –0.38; Cohen *d*=–0.90	
	Total	3.18 (0.71)	62	2.59 (0.69)		41 (66)		
	2	3.19 (0.73)	29	2.69 (0.64)		19 (65)		
	1	3.17 (0.71)	33	2.51 (0.74)		22 (67)		
**Depression^e^**							β=–.16; t_40.63_=–2.07; *P*=.04; 95% CI –0.32 to 0; Cohen *d*=–0.31	
	Total	0.95 (0.52)	62	0.82 (0.52)		39 (63)		
	2	1.02 (0.59)	29	0.92 (0.53)		18 (62)		
	1	0.89 (0.45)	33	0.73 (0.50)		21 (64)		
**Anxiety^f^**							β=–.21; t_39.2_=–1.88; *P*=.07; 95% CI –0.44 to 0.02; Cohen *d*=–0.28	
	Total	1.21 (0.78)	62	0.96 (0.72)		38 (61)		
	2	1.31 (0.86)	29	1.06 (0.82)		18 (62)		
	1	1.13 (0.72)	33	0.87 (0.61)		20 (61)		
**Well-being^g^**							β=.05; t_37.69_=0.32; *P*=.75; 95% CI –0.26 to 0.35; Cohen *d*=0.05	
	Total	4.97 (0.99)	62	4.98 (0.89)		38 (61)		
	2	4.82 (0.92)	29	4.69 (0.79)		18 (62)		
	1	5.10 (1.05)	33	5.25 (0.91)		20 (61)		
**Loneliness^h^**							β=–.09; t_39.57_=–1.04; *P*=.30; 95% CI –0.28 to 0.09; Cohen *d*=–0.16	
	Total	2.17 (0.61)	62	2.21 (0.55)		38 (61)		
	2	2.18 (0.63)	29	2.26 (0.56)		18 (62)		
	1	2.16 (0.60)	33	2.17 (0.56)		20 (61)		
**Self-concept clarity^i^**							β=.08; t_40.63_=0.68; *P*=.50; 95% CI –0.16 to 0.32; Cohen *d*=0.10	
	Total	3.16 (0.83)	62	3.16 (0.83)		39 (63)		
	2	3.27 (0.94)	29	3.17 (0.84)		18 (62)		
	1	3.06 (0.73)	33	3.20 (0.88)		21 (64)		
**Centrality of event^i^**							β=–.45; t_42.07_=–2.68; *P*=.01; 95% CI –0.78 to –0.11; Cohen *d*=–0.45	
	Total	3.72 (0.95)	62	3.24 (1.06)		38 (61)		
	2	3.72 (0.82)	29	3.24 (0.87)		18 (62)		
	1	3.72 (1.06)	33	3.24 (1.24)		20 (61)		
**Self-continuity^i^**							β=–.07; t_42.78_=–0.45; *P*=.66; 95% CI –0.41 to 0.26; Cohen *d*=0.08	
	Total	2.66 (0.88)	62	2.70 (0.94)		38 (61)		
	2	2.77 (0.76)	29	2.85 (0.93)		18 (62)		
	1	2.57 (0.97)	33	2.57 (0.95)		20 (61)		

^a^2: LIVIA 2.0; 1: LIVIA 1.

^b^Estimates of fixed effects.

^c^All the n values here correspond to 100% of the randomized sample.

^d^Grief symptoms were assessed using the Traumatic Grief Inventory.

^e^Depression symptoms were assessed using the Patient Health Questionnaire.

^f^Anxiety symptoms were assessed using the Generalized Anxiety Disorder-7.

^g^Well-being was assessed using the Flourishing Scale.

^h^Loneliness was assessed using the University of California, Los Angeles Loneliness Scale.

^i^Identity scales used were the Self-Concept Clarity Scale, Centrality of Event Scale, and Self-Continuity items.

### Treatment Satisfaction

Participants of the ITT sample reported overall high levels of satisfaction with the LIVIA programs (mean 3.18, SD 0.54; maximum score=4). There were however no significant differences between arms (t_32_=0.33; *P*=.75; mean_LIVIA 1_ 3.21, SD _LIVIA 1_ 0.50; mean_LIVIA 2.0_ 3.15 SD_LIVIA 2.0_ 0.59). Satisfaction scores across subdimensions were very similar: satisfaction with the theoretical content (mean 3.55, SD 0.43), satisfaction with the practical content (mean 3.46, SD 0.42), and satisfaction with the structure (mean 3.48, SD 0.49). None of these dimensions showed significant differences between arms (t_32_>0.03 <0.48, *P*>.63 <.93). Note that we conducted analyses on the ITT sample, as they are more conservative and comprise a larger sample.

### Stability of the Effects

Table 5 contains the post–follow-up effect sizes, as wells as means, and SDs of all outcome measures for the treatment groups 3 months after postintervention measurement. A total of 32 (52%) participants completed the follow-up measurement (ITT analyses) and 22 (36%) were included in the per-protocol analyses. The ITT analyses indicated that the effects were mostly stable at follow-up, except for self-concept clarity, which significantly improved at the within-group level, and loneliness and self-continuity, which showed a trend toward (further) improvement (but should be interpreted with caution given the limited sample size). The results of the per-protocol analyses, detailed in Multimedia Appendix 2, revealed similar results, with the exception that loneliness remained stable and self-concept clarity showed only a trend toward improvement.

**Table 5:** Stability of the effects from postintervention to follow-up in the intention-to-treat sample (n=32).

Domain and arm^a^		Follow-up			Postintervention to follow-up^b^ (n=32)
		Scores, mean (SD)	Participants, n (%)^c^		
**Grief^d^**				β=–.10; t_32.30_=–0.71; *P*=.48; 95% CI –0.40 to 0.19; Cohen *d*=–0.14	
	Total	2.46 (0.77)	32 (52)		
	2	2.64 (0.61)	15 (52)		
	1	2.30 (0.87)	17 (52)		
**Depression^e^**				β=–.07; t_27.78_=−0.83; *P*=.41; 95% CI –0.23 to 0.10; Cohen *d*=–0.13	
	Total	0.62 (0.53)	29 (47)		
	2	0.68 (0.52)	13 (45)		
	1	0.58 (0.56)	16 (49)		
**Anxiety^f^**				β=–.14; t_27.19_=−1.38; *P*=.18; 95% CI –0.34 to 0.07; Cohen *d*=–.18	
	Total	0.72 (0.70)	29 (47)		
	2	0.85 (0.88)	13 (45)		
	1	0.62 (0.53)	16 (49)		
**Well-being^g^**				β=.21; t_30.45_=1.09; *P*=.28; 95% CI –0.18 to 0.60; Cohen *d*=0.21	
	Total	5.17 (1.16)	29 (47)		
	2	4.76 (0.66)	13 (45)		
	1	5.51 (1.38)	16 (49)		
**Loneliness^h^**				β=–.16; t_28.12_=−1.79; *P*=.08; 95% CI –0.34 to 0.02; Cohen *d*=–0.27	
	Total	2.09 (0.64)	29 (47)		
	2	2.20 (0.62)	13 (45)		
	1	2.01 (0.66)	16 (49)		
**Self-concept clarity^i^**				β=.30; t_27.82_=2.66; *P*=.01; 95% CI 0.07 to 0.53; Cohen *d*=0.35	
	Total	3.54 (0.84)	29 (47)		
	2	3.50 (0.84)	13 (45)		
	1	3.58 (0.86)	16 (49)		
**Centrality of event^i^**				β=–.15; t_27.57_=−0.97; *P*=.34; 95% CI –0.47 to 0.17; Cohen *d*=–0.13	
	Total	3.13 (1.15)	29 (47)		
	2	3.26 (1.06)	13 (45)		
	1	3.03 (1.24)	16 (49)		
**Self-continuity^i^**				β=.34; t_28.19_=1.93; *P*=.06; 95% CI –0.02 to 0.70; Cohen *d*=0.35	
	Total	2.92 (1.04)	29 (47)		
	2	2.97 (0.99)	13 (45)		
	1	2.88 (1.11)	16 (49)		

^a^2: LIVIA 2.0; 1: LIVIA 1.

^b^The numbers corresponding to 100% of the randomized sample are found in Table 4.

^c^Estimates of fixed effects.

^d^Grief symptoms were assessed using the Traumatic Grief Inventory.

^e^Depression symptoms were assessed using the Patient Health Questionnaire.

^f^Anxiety symptoms were assessed using the Generalized Anxiety Disorder-7.

^g^Well-being was assessed using the Flourishing Scale.

^h^Loneliness was assessed using the University of California, Los Angeles Loneliness Scale.

^i^Identity scales used were the Self-Concept Clarity Scale, Centrality of Event Scale, and Self-Continuity items.

## Discussion

### Principal Findings

This study aimed to compare 2 WBIs for individuals with prolonged grief symptoms due to either death or romantic separation: LIVIA 1, an established program serving as the control condition and LIVIA 2.0, a newly developed program. This study demonstrated that both programs were effective in reducing grief and depression symptoms as well as the centrality of the loss and improving self-concept clarity. However, no effect was found for other outcomes. Moreover, no difference emerged between both programs’ efficacy, dropout rates, and satisfaction level.

Overall, this study demonstrated that both programs effectively reduced grief and depression symptoms, with large effect sizes for grief and medium effect sizes for depression symptoms. The benefits acquired during the programs were maintained 3 months after the intervention.

Beyond traditional symptom measures, the study also assessed identity-related concepts, recognizing that interpersonal loss can negatively impact self-concept and identity, which predicts prolonged grief symptoms [27,33]. Notably, 2 innovative findings emerged. First, the centrality of the loss to participants’ identities decreased following the programs; second, participants’ self-concept clarity improved significantly at follow-up. The centrality of the loss to one’s identity is associated with prolonged grief reactions [69]. Most cognitive therapies aim to alter narrative interpretations of traumatic events [70]. The results of this study indicate that both interventions reduced the dominance of the loss in participants’ identities and daily experiences, an effect that persisted posttest stage. This was also maintained 6 months later, demonstrating the sustainability of the programs’ impacts. This shift occurred even in LIVIA 1, which does not explicitly focus on identity processes, highlighting the flexibility of cognitive behavioral therapy interventions in addressing complex grief reactions. These interventions may enhance coping strategies, facilitate cognitive and emotional processing, and thus contribute to the reconstruction of identity after a loss (eg, [71]). In addition, this suggests that the programs might have helped participants normalize their grief and find new meanings in the loss, thereby reducing its overwhelming influence. This might have implications for the participants’ ability to reinvest in other aspects of life, contributing to an overall increase in resilience [72]. Given these observations, future research should explore the potential mediating role of decreased event centrality on grief symptoms using larger samples [27,72].

However, several outcomes, including well-being, loneliness, self-concept clarity, and self-continuity, were not significantly affected by the interventions. The programs might have lacked specific content regarding these outcomes to produce detectable effect sizes. For instance, loneliness showed a medium effect size improvement but was not statistically significant. Finally, self-concept clarity improved between posttest and follow-up stages. The interventions may have initiated a process of reflection and re-evaluation that takes time to manifest in tangible improvements in self-concept clarity. Participants might have needed additional time after intervention to internalize the changes and insights gained during the program, leading to a clearer and more stable sense of self over time [73,74]. However, these findings need replication given the small sample size.

There were no significant differences between LIVIA 1 and LIVIA 2.0 in outcomes or dropout rates, contrary to our hypothesis. This aligns with the generally limited differences found between various psychological interventions [75]. One possible explanation is that the innovations included in LIVIA 2.0 (ie, tailoring, increased interactivity, and the identity-focused module; refer to the study by Efinger et al [38]) may not have been sufficient to create a clear distinction between the programs. In addition, both programs were based on similar theoretical foundations, relying on cognitive behavioral therapy and the DPM of grief [34]. Finally, the limited power prevented the detection of small effects.

One significant innovation was the use of guidance on demand. This showed limited success, as there were only very few guidance requests, most of which concerned technical issues (refer to the study by Efinger et al [38]). This might explain the much higher dropout rate compared with Brodbeck et al [18] guided version of LIVIA 1 (37/62, 40% for this project vs 11% for theirs) However, a recent study on an unguided internet intervention for grieving adults during the COVID-19 pandemic showed much higher dropout rates (90%) [76]. Thus, the innovations implemented, particularly the automated emails and the possibility to ask for help if needed, might have boosted the retention rates, albeit to a lesser degree than weekly guidance would have. This reflects research on social support, which shows that it is most beneficial aspect is its perceived availability [77].

Completers showed high satisfaction with the theoretical content, practical content, and program structure, with no significant differences between the programs. This further suggests that the innovations integrated into LIVIA 2.0 did not have a substantial impact. Future research should further explore the factors contributing to participant satisfaction and retention, such as those proposed by Ritterband et al [78] for behavior change by WBIs. Although the differences between both programs might be small, the study’s statistical power was insufficient to detect such differences.

### Strengths and Limitations

This study compared 2 competing WBIs targeting prolonged grief symptoms after interpersonal loss, addressing a gap in comparative research on psychological interventions for grief [79]. To the best of our knowledge, this is the first study testing the efficacy of a grief WBI in French. The sample exhibited a wide diversity in terms of age, gender, type of loss, and employment type, effectively representing the population of people with prolonged grief symptoms. However, there was an overrepresentation of individuals with a university degree.

An important limitation of the study is its sample size. Initially, 264 participants were planned to detect small effect sizes and conduct moderation analyses but only 27 (44%) of the 62 participants were finally included in the per-protocol analyses, limiting the interpretability of the results as well as the risk of type II error. The small sample size was due to several factors: (1) the recruitment phase was limited by new requirements for web-based research in Switzerland [80]; (2) the eligibility criteria excluded many interested people (the most common exclusion reasons being already undergoing a treatment, the loss of a loved one having occurred <6 months ago, and moderate to acute suicidality; Figure 1); (3) the necessity of obtaining the original informed consent from every participant (with handwritten signature), despite the entire procedure being conducted on the web (Figure 1); (4) high access to psychotherapy in Switzerland (research indicates that, when given the choice, people often prefer traditional face-to-face psychotherapy [81]); and (5) the relatively small French-speaking population in Switzerland, while larger populations would be needed for specific sample recruitment. The small sample size limits statistical power, allowing only the detection of medium pre-post effect sizes. Therefore, the absence of effects on certain outcomes (eg, loneliness and well-being) and the lack of differences between the 2 interventions are inconclusive.

Another potential limitation is the large diversity of the participants (eg, in terms of age, type of loss, and time since the loss). Tailoring interventions to the specific needs of subsamples might be beneficial. Developing a more sophisticated specification algorithm could improve content adjustment according to participant profiles [82], potentially incorporating a therapeutic chatbot and artificial intelligence technologies [83].

As is common in eHealth trials, this study examined multiple outcomes, which increased the risk of type I error [84]. In addition, due to the limited sample size, we did not examine the influence of the extent to which participants used the intervention. Finally, the somewhat complex informed consent process requiring a handwritten signature might have discouraged some people from participating, potentially inducing bias in the sample selection.

### Clinical Implications

The results and observations from this study might provide valuable insights into clinical application. First, given the high attrition rate during the recruitment process (only 62/232, 26.7% of interested individuals were randomized), it is likely that recruitment could improve in a more naturalistic setting. Second, offering the program in a blended therapy setting, as commonly practiced in clinical environments [8], would allow customization of the timing of the content proposed by the LIVIA programs.

### Research Perspectives

In response to the high dropout rate, we are conducting interviews with participants who discontinued the program to understand their motives and derive strategies for better retention. In addition, a detailed analysis of browsing behaviors would enable us to understand how the program is used. This would provide reliable objective secondary data, allowing, for example, improvements in (differential) indication. To obtain more conclusive results, it is essential to gather a larger sample by recruiting participants from other French-speaking European countries. While technological advancements facilitate this process, heterogeneous regulations can complicate it [85]. Finally, adopting a co-design approach [86] could be beneficial for a better tailoring of the intervention. This could involve organizing focus groups with the target population and conducting interviews with key stakeholders, such as grief therapists.

## Appendix

Multimedia Appendix 1 Participant information and informed consent form.

Multimedia Appendix 2 Comparison of efficacy between LIVIA 2.0 and LIVIA 1, and stability of effects with the per-protocol analyses.

Multimedia Appendix 3 CONSORT-eHEALTH checklist (V 1.6.1).

## Abbreviations

DPM: dual process model
ITT: intent-to-treat
REDCap: Research Electronic Data Capture
SIDAS: Suicidal Ideation Attributes Scale
WBI: web-based intervention

## Data Availability

The dataset generated and analyzed during this study is available in the SWISSUbase repository [87].
