# Supplementary material for: A French-Language Web-Based Intervention Targeting Prolonged Grief Symptoms in People Who Are Bereaved and Separated: Randomized Controlled Trial
Source: JMIR Form Res. 2024 Oct 16;8:e57294. doi: 10.2196/57294 (PMC11525088; doi:10.2196/57294)
Supplement: Multimedia Appendix 1 [file formative_v8i1e57294_app1.pdf]

Demande de participation à un projet de recherche médical :

Évaluation de deux programmes d'auto-soutien en ligne (LIVIA 1 et LIVIA 2.0) pour adultes présentant des difficultés à gérer la perte d'une personne proche (par décès ou séparation/divorce)

Madame, Monsieur,

Nous vous proposons de participer à notre projet de recherche.

Votre participation est entièrement libre. Toutes les données collectées dans le cadre de ce projet sont soumises à des règles strictes en matière de protection des données.

Le projet de recherche est mené par les Drs Anik Debrot et Valentino Pomini. Nous vous en communiquerons les résultats si vous le souhaitez.

Avec ce document, nous vous présenterons les éléments essentiels**. En cas de questions ou doutes, nous sommes disponibles sur** psyconsultonline@unil.ch **ou au +41 21 692 32 39**. Pour vous proposer d'ores et déjà un aperçu du projet, voici les points clés à retenir. Vous trouverez à la suite des informations complémentaires plus détaillées.

Pourquoi menons-nous ce projet de recherche ?

- En présence de difficultés à faire face à la perte d'une personne proche, des interventions par internet existent depuis quelques années dans le but de diminuer les symptômes de deuil et améliorer le bien-être.
- Notre projet de recherche vise à étudier si l'efficacité présentée par une nouvelle intervention par internet francophone développée par nos soins (LIVIA 2.0) est équivalente ou supérieure à une intervention ayant déjà démontré son efficacité (LIVIA 1).

Que dois-je faire si j'accepte de participer ? – Que se passe-t-il pour moi en cas de participation ?

- Forme de la participation : Si vous acceptez de participer à notre projet, vous aurez accès à une des deux interventions par internet et pourrez suivre les 10 séances sur une durée de 3 mois.
  Déroulement pour les participants : Nous vérifierons premièrement si vous remplissez les critères de participation à l'aide d'un formulaire en ligne. Si vous remplissez les critères de sélection, vous pourrez télécharger le document présent, le lire et remplir, signer **MANUELLEMENT** (ceci est nécessaire d'un point de vue légal), scanner et nous le remettre, soit en le téléversant à l'aide du lien suivant : https://drive.switch.ch/index.php/s/MAS3yUsJNZY0eSL, soit en nous le renvoyant par courrier postal (nous vous envoyons volontiers alors une enveloppe-réponse préaffranchie si vous nous communiquez votre adresse postale à cette adresse e-mail : psyconsultonline@unil.ch). **A ce stade, vous pourrez poser toutes les questions à l'équipe de recherche (soit par e-mail : psyconsultonline@unil.ch**

ou par téléphone : 021 692 32 39) ou en discuter avec toutes les personnes que vous souhaitez (famille, amis, professionnel·le·s de la santé, etc.) avant de vous décider à participer. Si vous vous décidez à participer, nous vous demandons d'imprimer, signer MANUELLEMENT et nous renvoyer par e-mail ce document.
Quand il nous sera parvenu, nous vous enverrons par e-mail le lien pour répondre aux questionnaires à remplir avant le début de l'intervention. Une fois que vous aurez rempli les questionnaires, nous vous enverrons le lien pour créer votre compte et accéder librement au programme d'intervention en ligne. Le choix du programme LIVIA 1 ou LIVIA 2.0 sera fait de manière aléatoire et la probabilité d'attribution à l'un ou l'autre des programmes est d'une chance sur deux ($P = \frac{1}{2}$). La totalité du programme peut être accomplie en 10 à 12 semaines, à raison d'environ 1 séance par semaine. Trois mois après avoir commencé le programme, nous vous transmettrons une série de questionnaires en ligne – quelque soit votre avancée dans le programme. Enfin, encore trois mois plus tard, une dernière série de questionnaires vous sera envoyée. Les résultats obtenus aux différents questionnaires nous permettront d'évaluer scientifiquement l'efficacité du nouveau programme. Sachez également qu'après la fin de votre participation à l'étude, vous garderez l'accès au programme pour au moins un an (vous serez averti·e par e-mail quand nous fermerons la plateforme après ce délai).

- Durée : 3 mois pour faire le programme + 3 mois plus tard, remplir les derniers questionnaires

Quels sont les bénéfices et les risques liés à la participation au projet ?

Bénéfices pour les participant·e·s

- La participation vous donne accès gratuitement à une intervention d'auto-soutien en ligne créée par des professionnels de la santé. Nous nous attendons à ce que votre état s'améliore après avoir réalisé le programme. Néanmoins, nous ne pouvons pas vous garantir que vous retirerez un bénéfice direct en prenant part à cette étude.
- Par votre participation, vous contribuez à aider les futures personnes rencontrant des problèmes similaires.

Risques et contraintes

- Il n'y a pas d'indications que votre participation soit liée à un risque, mis à part celui de raviver certains souvenirs douloureux durant la réalisation du programme. Cependant, nous avons mis en place diverses procédures pour réduire tout risque potentiel pour vous.

En apposant votre signature à la fin du document, vous certifiez en avoir compris tout le contenu et consentir librement à prendre part au projet.

Information détaillée

1. Objectif du projet et sélection des participant·e·s

Nous effectuons cette étude pour évaluer l'efficacité de deux programmes d'auto-soutien en ligne. Plus précisément, nous visons à comparer les effets des programmes LIVIA 1 et LIVIA 2.0 auprès de personnes ayant vécu d'une perte interpersonnelle qui les fait souffrir. Ce projet contribuera à l'avancée des connaissances sur les programmes psychologiques d'auto-soutien par internet et à promouvoir des méthodes d'intervention novatrices, peu disponibles actuellement en français.

Nous vous sollicitons car la participation est ouverte à toutes les personnes qui présentant des difficultés à faire face suite à la perte d'une personne proche (par décès, séparation ou divorce).

2. Informations générales sur le projet

- LIVIA est un programme par internet, développé à l'Université de Berne en allemand, destiné aux personnes ayant des difficultés à surmonter la perte de leur partenaire (par séparation/divorce ou deuil). Autant sa version allemande que la version française traduite par notre équipe a démontré son efficacité pour réduire la détresse liée à la perte d'une personne proche.
- Le programme LIVIA 2.0 est un nouveau programme d'auto-soutien par internet pour les personnes éprouvant des difficultés à surmonter la perte d'une personne proche.
- Les deux programmes LIVIA sont composés de dix séances et contiennent des informations sur le processus de la perte, des tâches de réflexion personnelle et des propositions d'activités. Ces interventions se basent sur des éléments de thérapie cognitivo-comportementale.
- L'étude est basée à Lausanne en Suisse et se déroulera en ligne. Elle durera 18 mois pendant lesquels nous aimerions recruter au moins 234 participant·e·s (117 pour LIVIA 1 et 117 pour LIVIA 2.0). Chaque programme d'auto-soutien dure 2 mois et demi à 3 mois. Tou·te·s les participant·e·s auront accès directement au programme ; il sera décidé aléatoirement à quel programme chaque participant·e sera attribué·e. La probabilité d'attribution à l'un ou l'autre des programmes est d'une chance sur deux ($P = \frac{1}{2}$).
- Nous effectuons cette étude dans le respect des prescriptions de la législation suisse et des normes éthiques internationales. L'étude a été approuvée par les autorités compétentes (Commission cantonale d'éthique de la recherche sur l'être humain, Vaud et Swissmedic).
- Vous trouverez aussi un descriptif de l'étude sur le site Internet de l'Office fédéral de la santé publique : www.kofam.ch.

3. Déroulement pour les participant·e·s

Nous vérifierons premièrement si vous remplissez les critères de participation à l'aide du formulaire en ligne que vous venez de remplir. Toutes les personnes remplissant les critères de participation arriveront sur une page où ils·elles pourront télécharger le présent formulaire de consentement, avec toutes les informations sur l''étude. **A ce stade, vous pourrez poser toutes les questions à l'équipe de recherche (soit par e-mail : psyconsultonline@unil.ch ou par téléphone : 021 692 32 39) ou en discuter avec toutes les personnes que vous souhaitez (famille, amis, professionnel·le·s de la santé, etc.) avant de vous décider à participer.**

Si vous vous décidez à participer, nous vous demandons d'imprimer, signer MANUELLEMENT (ceci est nécessaire d'un point de vue légal) et nous remettre ce document, soit en le téléversant à l'aide du lien suivant : https://drive.switch.ch/index.php/s/MAS3yUsJNZY0eSL, soit en nous le renvoyant par courrier postal (nous vous envoyons volontiers alors une enveloppe-réponse préaffranchie si vous nous communiquez votre adresse postale à cette adresse e-mail : psyconsultonline@unil.ch**).**

Une fois qu'il nous sera parvenu, nous vous enverrons par e-mail un lien unique pour répondre aux questionnaires à remplir avant le début de l'intervention, ce lien permettra que vos données restent codées et donc non-identifiables. Une fois que vous aurez rempli les questionnaires, nous vous enverrons le lien et vos accès personnels pour accéder librement au programme d'intervention en ligne (le choix du programme LIVIA 1 ou LIVIA 2.0 sera fait de manière aléatoire). La totalité du programme peut être accomplie en 10 à 12 semaines, à raison d'environ 1 module par semaine. Une fois le programme terminé, nous vous transmettrons une série de questionnaires en ligne. Enfin, trois mois après avoir fini le programme, une dernière série de questionnaires en ligne vous sera envoyée. Les résultats obtenus aux différents questionnaires nous permettront d'évaluer scientifiquement l'efficacité de chaque programme.

Le Tableau ci-dessous indique les détails du déroulement pour les participant·e·s.

**Point de temps**	**Étape**	**Procédure détaillée – qu'est-ce qui est fait ?**	**Temps estimé**
	**Inscription à l'étude**	Les personnes intéressées par l'étude peuvent consulter la page d'accueil du projet. Elles peuvent s'inscrire à l'aide d'un lien les dirigeant vers un formulaire qui permettra de vérifier automatiquement les critères de participation de base.	5 minutes
	**Formulaire de consentement**	S'il/elle·s sont éligibles, les participant·e·s peuvent télécharger la fiche d'information et le formulaire de consentement. S'il/elle·s l'approuvent, il/elle·s le signeront et nous le renverront par e-mail (s'il/elle·s ont des questions sur l'étude, les participant·e·s pourront nous contacter via l'e-mail ou téléphone).	20 minutes
Début de l'étude<br>T0	**Évaluation pré-test**<br>Compléter les questionnaires en ligne	Après avoir reçu le formulaire signé, nous enverrons aux participant·e·s un lien individuel vers la plateforme de questionnaire RedCap. Cela nous permettra d'identifier le/la participant·e sans qu'il/elle ait à fournir d'informations identifiables. Les participant·e·s seront ensuite invité·e·s à remplir les questionnaires en ligne du pré-test (T0).	15-20 minutes
Dès T0 + 1 jour<br>Pendant 12 semaines	**Phase d'intervention**	<u>Une fois les questionnaires complétés, le lendemain les participant·e·s reçoivent le lien vers le site internet de l'intervention et commence le programme ce jour-là.</u> Le choix du programme LIVIA 1 ou LIVIA 2.0 sera fait de manière aléatoire. Pour des raisons scientifiques, les participant·e·s ne sont pas informé·e·s de quel programme leur a été attribué. Les participant·e·s peuvent choisir un mot de passe et l'administration de l'intervention d'auto-soutien sur 12 semaines peut commencer.	Environ 45-60 minutes par semaine (pendant 10-12 semaines)
Après la fin de l'intervention<br>T1	**Fin de l'intervention**<br>Compléter les questionnaires en ligne	Après la fin de l'intervention (environ 12 semaines), nous demandons à tou·te·s les participant·e·s de remplir les questionnaires en ligne post-intervention.	15-20 minutes
Six mois après le début de l'intervention<br>T2	**Test de la stabilité des effets**<br>(trois mois après la fin de l'intervention)	Pour tester la stabilité des effets de l'intervention, les participant·e·s seront contacté·e·s par e-mail et il leur sera demandé de remplir une seconde fois les questionnaires en ligne post-intervention.	15-20 minutes

Pendant le programme, les participant·e·s recevront des e-mails automatisés dans deux situations : a) quand une nouvelle séance sera disponible, et b) si le/la participant·e ne s'est pas connecté·e au programme depuis au moins 7 jours.

A l'issue du programme d'auto-soutien, nous vous demanderons si vous avez débuté une psychothérapie ou un traitement médicamenteux psychotrope (par ex. antidépresseur ou anxiolytique) parallèlement au programme.

Les participant·e·s présentant un risque suicidaire aigu ne peuvent pas utiliser le programme d'auto-soutien tant que leur situation n'est pas stabilisée et leur risque à un niveau faible. Si le risque est élevé, nous fournirons des informations sur les possibilités d'aide en Suisse romande, Belgique,

Canada et France. Si le risque est modéré, nous procéderons à une évaluation plus poussée par téléphone.
Il se peut que nous devions vous retirer de l'étude avant le terme prévu. Cette situation peut se produire si votre vie est menacée.
Sachez également qu'après la fin de votre participation à l'étude, vous garderez accès au programme pour au moins un an (vous serez averti par e-mail quand nous fermerons la plateforme après ce délai).

4. Bénéfices pour les participant·e·s

La participation vous donne accès gratuitement à une intervention d'auto-soutien en ligne qui a été élaborée par des professionnels de la santé. Nous nous attendons à ce que votre état s'améliore suite à la passation de chaque programme. Néanmoins, nous ne pouvons pas vous garantir que vous retirerez un bénéfice direct en prenant part à cette étude. Cependant, les données récoltées dans ce projet permettront à d'autres personnes se trouvant dans la même situation que vous de bénéficier d'un soutien dans le futur grâce aux connaissances acquises.

5. Caractère facultatif de la participation et obligations

Votre participation est entièrement libre. Si vous choisissez de ne pas participer ou si vous choisissez de participer et revenez sur votre décision pendant le déroulement de l'étude, vous n'aurez pas à vous justifier. Cela ne changera rien à votre accès au programme LIVIA. En effet, votre accès restera toujours valide suite à votre rétractation de participation à l'étude.

Si vous choisissez de participer à l'étude, vous serez tenu·e :

- d'effectuer les modules du programme d'auto-soutien par internet et de répondre à des questionnaires à trois reprises. Une partie des participant·e·s répondront également au monitoring hebdomadaire durant la durée du programme ; par monitoring, nous entendons 5 brèves questions qui concernent votre ressenti et état du moment ;
- d'informer l'investigatrice principale, par e-mail (psyconsultonline@unil.ch), de tout nouveau symptôme indicatif d'une crise et tout changement dans votre état, notamment en présence de pensées suicidaires ;

> Si vous avez des pensées suicidaires, vous trouvez ci-dessous les numéros que vous pouvez composer ; ils sont disponibles 24h/24, 7 jours sur 7 :
> **Suisse** : Main tendu, ligne 143 ;
> **Belgique** : Télé-Accueil, numéro 107 ou Prévention Suicide, numéro 0800 32 123 ;
> **Canada** : Le service d'intervention téléphonique 1 866 APPELLE ou 1 866 277 3553 ;
> **France** : Suicide Écoute, numéro 01 45 39 40 00.

- d'informer l'investigatrice principale, par e-mail (psyconsultonline@unil.ch), de tout traitement ou thérapie concomitant, prescrit par un autre médecin ; de l'informer également de tous les médicaments que vous prenez contre la dépression ou l'anxiété.

6. Risques et contraintes pour les participant·e·s

La passation des questionnaires ainsi que la complétion du programme représentent un investissement temporel pour les participant·e·s. Théoriquement et sur la base des recherches faites jusqu'à présent, vous ne devez pas vous attendre à des effets négatifs ou à une péjoration de votre état en prenant part à l'étude sur le programme d'auto-soutien. Il n'y a pas d'indications que votre participation soit liée à un risque, mis à part celui de raviver des souvenirs douloureux durant la réalisation du programme. Cependant, nous avons mis en place diverses procédures pour réduire davantage tout risque potentiel ou aversif pour vous.

7. Alternatives

Vous n'êtes pas tenu·e de participer à l'étude. Si vous décidez de ne pas y prendre part, il sera toujours possible de vous tourner vers d'autres moyens pour trouver du soutien dans votre réaction de deuil ou de vous inscrire à cette étude dans un temps futur. Ce soutien peut être obtenu auprès de groupes d'entraide, d'aumôneries ou encore de lieux de conseils. Pour les personnes qui souffrent intensivement de la perte, une psychothérapie pour traiter les réactions de deuil compliqué ou autres troubles peut s'avérer utile.
Si vous deviez décider d'entamer une psychothérapie avant, pendant ou après votre participation à l'étude, nous vous conseillerions volontiers au sujet des différentes possibilités qui s'offrent à vous. Même si vous êtes amené·e à vous retirer du programme, pour une quelconque raison, vous pourrez continuer à utiliser le programme d'auto-soutien en dehors de l'étude. De plus, vous garderez accès au programme pour au moins un an (vous serez averti·e par e-mail quand nous fermerons la plateforme après ce délai).

8. Résultats

L'étude permet d'obtenir différents résultats :
1. des résultats individuels qui vous concernent directement,
2. les résultats définitifs objectifs de l'étude dans son ensemble.

1. L'investigatrice ou l'investigateur vous avisera pendant l'étude de toute nouvelle découverte importante vous concernant. Vous serez informé·e par écrit ; vous pourrez par la suite à nouveau décider si vous souhaitez poursuivre votre participation à l'étude.

2. L'investigatrice principale peut vous faire parvenir, à l'issue de l'étude, une synthèse des résultats globaux.

9. Confidentialité des données et des échantillons

Pour les besoins de l'étude, nous enregistrerons vos données personnelles (e-mail, nom, prénom, date de naissance et no de téléphone). Ces données nous serons transmises par vos soins par le présent formulaire envoyé par e-mail. Elles seront soigneusement tenues séparément de toutes les autres données de l'étude.

Dans le cas d'une publication, les données de l'ensemble des participants ne vous seront pas imputables en tant que personne. Aucune donnée identifiante n'apparaîtra jamais sur Internet ou dans une publication. Les journaux et organismes de financement peuvent demander le partage sur un site dédié des données à l'origine des publications ('Open Data'). Cette démarche de partage des données permet la validation des résultats publiés (reproductibilité), de cumuler les données de différentes recherches et plus généralement l'utilisation des données par d'autres chercheuse et chercheurs. Dans le cas d'un tel partage, vos données seront toujours codées de façon à ce qu'il ne soit pas possible de remonter à votre identité. En cas de retrait de votre consentement à participer à ce projet, les données déjà partagées ne pourront pas être retirées. Vous devez être d'accord avec cela si vous participez à l'étude.

L'accès au programme d'auto-soutien est protégé par un mot de passe, la transmission de vos données par le biais d'internet se fait de manière chiffrée, et donc protégée.

Si vous nous donnez un autre accord à part (cf. fin du document), vos données pourront être utilisées pour d'autres recherches futures sur des thématiques similaires. À nouveau, dans ce cas, seules vos données codées seront utilisées.

9.1. Traitement et codage des données

Dans le cadre de cette étude, des données relatives à votre personne et à votre santé sont recueillies et traitées, en partie de manière automatisée. Ces informations sont codées au moment

du relevé. Le codage signifie que toutes les données permettant de vous identifier (nom, date de naissance, etc.) sont remplacées par un code. Il n'est pas possible de relier les données à votre personne sans le code, qui reste en permanence au sein de l'Université de Lausanne, sous les plus hauts standards de sécurité (serveur NAS).
Seul un nombre limité de personnes peut consulter vos données sous une forme non codée, et ce, exclusivement afin de pouvoir accomplir des tâches nécessaires au déroulement de l'étude. Ces personnes sont strictement tenues au secret professionnel. En tant que participant·e, vous avez le droit de consulter vos données.

9.2. Protection des données

Toutes les directives relatives à la protection des données sont rigoureusement respectées. Il est possible que vos données doivent être transmises sous forme codée, par exemple pour une publication, et qu'elles puissent être mises à la disposition d'autres chercheur·e ·s.

9.3. Droit de consultation dans le cadre d'inspections

L'étude peut faire l'objet d'inspections. Celles-ci peuvent être effectuées par la commission d'éthique compétente, ou par l'instance de monitoring mandatée à ce propos, en l'occurrence le Centre de Recherche Clinique du Centre Hospitalier Universitaire Vaudois. L'investigatrice doit alors communiquer vos données pour les besoins de ces inspections. Toutes les personnes impliquées sont tenues au plus strict secret professionnel.

10. Retrait du projet

Vous pouvez à tout moment vous retirer de l'étude si vous le souhaitez. Cependant, les données recueillis jusque-là pourront encore être analysés sous forme codée.

11. Dédommagement

Si vous participez à cette étude, vous ne recevrez pour cela aucune compensation, hormis l'accès gratuit au programme d'auto-soutien en ligne.

12. Responsabilité

En cas de dommages ou de lésions liés à la présente étude, la responsabilité de l'Université de Lausanne permet d'obtenir une indemnisation, à l'exception des réclamations qui découlent d'une faute ou d'une négligence grave.

13. Financement

L'étude est financée par l'Université de Lausanne ainsi que par le Fond National Suisse de la Recherche Scientifique (projet no 100014_182840).

14. Interlocuteur(s)

Vous pouvez à tout moment poser des questions au sujet de l'étude. En cas de doutes, de craintes ou d'urgences présentement, ou pendant ou après l'étude, vous pouvez vous adresser à l'un des interlocuteurs suivants :

- *Dr. Phil, Anik Debrot, Institut de Psychologie, Université de Lausanne, Géopolis, 1015 Lausanne. E-mail : psyconsultonline@unil.ch, Tél : +41 21 392 32 39.*

- *Prof. Valentino Pomini, Institut de Psychologie, Université de Lausanne, Géopolis, 1015 Lausanne. E-mail : psyconsultonline@unil.ch, Tél : +41 21 692 32 77.*

- *Maya Kheyar, Institut de Psychologie, Université de Lausanne, Géopolis, 1015 Lausanne. e-mail : psyconsultonline@unil.ch, Tél : +41 21 692 32 56*

Déclaration de consentement

Déclaration de consentement écrite pour la participation à une étude clinique
Veuillez lire attentivement ce formulaire. N'hésitez pas à poser des questions lorsque vous ne comprenez pas quelque chose ou que vous souhaitez avoir des précisions. Votre consentement écrit est nécessaire pour participer au projet.

**Numéro BASEC du projet de recherche :**	2021-D0086
**Titre (scientifique et usuel) :**	Évaluation de deux programmes d'auto-soutien en ligne (LIVIA 1 et LIVIA 2.0) pour adultes présentant des difficultés à gérer la perte d'une personne proche (par décès ou séparation/divorce)
**Institution responsable (promoteur et adresse complète) :**	Valentino Pomini et Anik Debrot, Institut de Psychologie, Université de Lausanne, Géopolis, 1015 Lausanne
**Lieu de réalisation :**	Étude par internet, basée à Lausanne
**Investigateur responsable sur le site :** Nom et prénom en caractères d'imprimerie :	Anik Debrot
**Participant / Participante :** Nom et prénom en caractères d'imprimerie :	
Date de naissance :	
No de téléphone :	
Adresse e-mail :	

- Je déclare avoir été informé·e, par l'investigatrice soussigné·e par écrit des objectifs et du déroulement de l'étude mettant en œuvre ainsi que des avantages et des inconvénients possibles et des risques éventuels.
- Je prends part à cette étude de façon volontaire et j'accepte le contenu de la feuille d'information qui m'a été remise. J'ai eu suffisamment de temps pour prendre ma décision.
- J'ai reçu les réponses aux éventuelles questions que j'ai posées en relation avec ma participation à cette étude. Je conserve la feuille d'information et reçois une copie de ma déclaration de consentement.
- J'accepte que les spécialistes compétents du promoteur de ce projet, de la commission d'éthique compétente (CER-VD), de Swissmedic et de l'instance de monitoring du Centre de Recherche Clinique (CRC) situé au Centre Hospitalier Universitaire Vaudois (CHUV) puissent consulter mes données non codées afin de procéder à des contrôles et des inspections, à condition toutefois que la confidentialité de ces données soit strictement assurée.
- Je serai informé·e des résultats et/ou de toute découverte importants ayant un lien avec ma santé.

- J’ai compris que mes données seront partagées en open data et qu’en cas de retrait de mon consentement, celles-ci ne pourront pas être retirées.
- Je peux, à tout moment et sans avoir à me justifier, révoquer mon consentement à participer à l’étude, sans que cette décision n’ait de répercussions défavorables sur la suite de ma prise en charge. Les données qui ont été recueillies jusque-là seront cependant analysées dans le cadre de l’étude.
- Je suis conscient·e que les obligations mentionnées dans la feuille d’information destinée aux participant·e·s doivent être respectées pendant toute la durée de l’étude. La direction de l’étude peut m’en exclure à tout moment dans l’intérêt de ma santé.

Lieu, date	Signature **MANUSCRITE** du/de la participant·e

Merci de nous remettre ce document signé, soit en le téléversant à l’aide du lien suivant : https://drive.switch.ch/index.php/s/MAS3yUsJNZY0eSL, soit en nous le renvoyant par courrier postal (nous vous envoyons volontiers alors une enveloppe-réponse préaffranchie si vous nous communiquez votre adresse postale à cette adresse e-mail : psyconsultonline@unil.ch).

Adresse :

Dr. Anik Debrot
Institut de Psychologie
Université de Lausanne
Géopolis
1015 Lausanne
