# Supplementary material for: A French-Language Web-Based Intervention Targeting Prolonged Grief Symptoms in People Who Are Bereaved and Separated: Randomized Controlled Trial
Source: JMIR Form Res. 2024 Oct 16;8:e57294. doi: 10.2196/57294 (PMC11525088; doi:10.2196/57294)
Supplement: Multimedia Appendix 2 [file formative_v8i1e57294_app2.docx]

**A French-language Web-based intervention targeting prolonged grief symptoms in bereaved and separated people: A randomized controlled trial**

**Multimedia Appendix 2**

Anik Debrot^1*^, Liliane Efinger^1^, Maya Kheyar^1^, Valentino Pomini^1^, & Laurent Berthoud^1^

^1^ Institute of Psychology, Faculty of Social and Political Sciences, University of Lausanne, Lausanne, Switzerland

*Table S1.* Comparison of efficacy between LIVIA 2.0 and LIVIA 1, and stability of effects with the Intention-to-treat analyses.

|  | Pre-treatment | | Post-treatment | | Follow-up | | Pre-post within group^a^ | Between group^a^ | Time x treatment^a^ | Post-follow-up^a^ (N=22) |
| --- | --- | --- | --- | --- | --- | --- | --- | --- | --- | --- |
| Domain | *M(SD)* | *n (%)* | *M(SD)* | *n (%)* | *M(SD)* | *n (%)* | *β, t(df), p,* [95% CI], d_cohen_ | *β, t(df), p,* [95% CI], d_cohen_ | *β, t(df), p,* [95% CI] | *β, t(df), p,* [95% CI], d_cohen_ |
| Grief  LIVIA 2.0  LIVIA 1 | 3.18(0.71)  3.19(0.73)  3.17(0.71) | 62 (100%)  29 (100%)  33 (100%) | 2.59(0.69)  2.69(0.64)  2.51(0.74) | 41 (66%)  19 (65%  22 (67%) | 2.46(0.77)  2.64(0.61)  2.30(0.87) | 32 (52%)  15 (52%)  17 (52%) | *β*=-.64, *t*(44.07)=-4.94, *p*<.001, [-.90; -.38], *d*=-.90 | *β*=.02, *t*(77.64)=.14, *p*=.89, [-.33; .38] | *β*=.21, *t*(44.18)=1.10, *p*=.28, [-.17; .59] | *β*=-.10, *t*(32.30)=-.71, *p*=.48, [-.40; .19], *d*=-.14 |
| Depression  LIVIA 2.0  LIVIA 1 | 0.95(0.52)  1.02(0.59)  0.89(0.45) | 62 (100%)  29 (100%)  33 (100%) | 0.82(0.52)  0.92(0.53)  0.73(0.50) | 39 (63%)  18 (62%)  21 (64%) | 0.62(0.53)  0.68(0.52)  0.58(0.56) | 29 (47%)  13 (45%)  16 (49%) | *β*=-.16, *t*(40.63)=-2.07, *p*=.04, [-.32; .00], *d*=-.31 | *β*=.13, *t*(70.51)=.99, *p*=.32, [-.13; .39] | *β*=.01, *t*(40.72)=.07, *p*=.94, [-.22; .24] | *β*=-.07, *t*(27.78)=-.83, *p*=.41, [-.23; .10], *d*=-.13 |
| Anxiety  LIVIA 2.0  LIVIA 1 | 1.21(0.78)  1.31(0.86)  1.13(0.72) | 62 (100%)  29 (100%)  33 (100%) | 0.96(0.72)  1.06(0.82)  0.87(0.61) | 38 (61%)  18 (62%)  20 (61%) | 0.72(0.70)  0.85(0.88)  0.62(0.53) | 29 (47%)  13 (45%)  16 (49%) | *β*=-.21, *t*(39.2)=-1.88, *p*=.07, [-.44; .02], *d*=-.28 | *β*=.17, *t*(68.92)=.90, *p*=.37, [-.21; .56] | *β*=-.001, *t*(19.13)=-.01, *p*=.99, [-.33; .33] | *β*=-.14, *t*(27.19)=-1.38, *p*=.18, [-.34; .07], *d*=-.18 |
| Well-being  LIVIA 2.0  LIVIA 1 | 4.97(0.99)  4.82(0.92)  5.10(1.05) | 62 (100%)  29 (100%)  33 (100%) | 4.98(0.89)  4.69(0.79)  5.25(0.91) | 38 (61%)  18 (62%)  20 (61%) | 5.17(1.16)  4.76(0.66)  5.51(1.38) | 29 (47%)  13 (45%)  16 (49%) | *β*=.05, *t*(37.69)=.32, *p*=.75, [-.26; .35], *d*=.05 | *β*=-.27, *t*(68.46)=-1.12, *p*=.27, [-.77; .22] | *β*=-.07, *t*(37.60)=-.31, *p*=.76, [-.51; .38] | *β*=.21, *t*(30.45)=1.09, *p*=.28, [-.18; .60], *d*=.21 |
| Loneliness  LIVIA 2.0  LIVIA 1 | 2.17(0.61)  2.18(0.63)  2.16(0.60) | 62 (100%)  29 (100%)  33 (100%) | 2.21(0.55)  2.26(0.56)  2.17(0.56) | 38 (61%)  18 (62%)  20 (61%) | 2.09(0.64)  2.20(0.62)  2.01(0.66) | 29 (47%)  13 (45%)  16 (49%) | *β*=-.09, *t*(39.57)=-1.04, *p*=.30, [-.28; .09], *d*=-.16 | *β*=-.02, *t*(69.81)=1.36, *p*=.89, [-.28; .32] | *β*=.15, *t*(39.49)=1.10, *p*=.28, [-.12; .42] | *β*=-.16, *t*(28.12)=-1.79, *p*=.08, [-.34; .02], *d*=-.27 |
| Self-concept clarity  LIVIA 2.0  LIVIA 1 | 3.16(0.83)  3.27(0.94)  3.06(0.73) | 62 (100%)  29 (100%)  33 (100%) | 3.16(0.83)  3.17(0.84)  3.20(0.88) | 39  18  21 | 3.54(0.84)  3.50(0.84)  3.58(0.86) | 29 (47%)  13 (45%)  16 (49%) | *β*=.08, *t*(40.63)=.68, *p*=.50, [-.16; .32], *d*=.10 | *β*=.21, *t*(69.17)=.97, *p*=.33, [-.22; .63] | *β*=-.14, *t*(40.70)=-.81, *p*=.42, [-.49; .21] | *β*=.30, *t*(27.82)=2.66, *p*=.01, [.07; .53], *d*=.35 |
| Centrality of event  LIVIA 2.0  LIVIA 1 | 3.72(0.95)  3.72(0.82)  3.72(1.06) | 62 (100%)  29 (100%)  33 (100%) | 3.24(1.06)  3.24(0.87)  3.24(1.24) | 38 (61%)  18 (62%)  20 (61%) | 3.13(1.15)  3.26(1.06)  3.03(1.24) | 29 (47%)  13 (45%)  16 (49%) | *β*=-.45, *t*(42.07)=-2.68, *p*=.01, [-.78; -.11], *d*=-.45 | *β*=.01, *t*(73.36)=-.02, *p*=.98, [-.51; .50] | *β*=.05, *t*(41.97)=.20, *p*=.84, [-.44; .54] | *β*=-.15, *t*(27.57)=-.97, *p*=.34, [-.47; .17], *d*=-.13 |
| Self-continuity  LIVIA 2.0  LIVIA 1 | 2.66(0.88)  2.77(0.76)  2.57(0.97) | 62 (100%)  29 (100%)  33 (100%) | 2.70(0.94)  2.85(0.93)  2.57(0.95) | 38 (61%)  18 (62%)  20 (61%) | 2.92(1.04)  2.97(0.99)  2.88(1.11) | 29 (47%)  13 (45%)  16 (49%) | *β*=-.07, *t*(42.78)=-.45, *p*=.66, [-.41; .26], *d*=.08 | *β*=.20, *t*(75.86)=.89, *p*=.37, [-.25; .66] | *β*=.10, *t*(42.66)=.43, *p*=.67, [-.38; .59] | *β*=.34, *t*(28.91)=1.93, *p*=.06, [-.02; .70], *d*=.35 |

*Note*. ITT = Intention to treat. Grief symptoms were assessed by using the Traumatic Grief Inventory; Depression symptoms were assessed by using the Patient Health Questionnaire (PHQ-9); Anxiety symptoms were assessed by using the General Anxiety Disorder-7 (GAD-7); Well-being was assessed by using the Flourishing Scale; Loneliness was assessed by using the UCLA Loneliness Scale; Identity scales used were the Self-Concept Clarity Scale, Centrality of Event Scale, and Self-Continuity items. ^a^ Estimates of fixed effects

*Table S2.* Comparison of efficacy between LIVIA 2.0 and LIVIA 1, and stability of effects with the Per-Protocol Analyses.

|  | Pre-treatment | | Post-treatment | | Follow-up | | Pre-post within group^a^ | Between group^a^ | Time x treatment^a^ | Post-follow-up^a^ |
| --- | --- | --- | --- | --- | --- | --- | --- | --- | --- | --- |
| Domain | *M(SD)* | *n* | *M(SD)* | *n* | *M(SD)* | *n* | *β, t(df), p,* [95% CI], d_cohen_ | *β, t(df), p,* [95% CI], d_cohen_ | *β, t(df), p,* [95% CI] | *β, t(df), p,* [95% CI], d_cohen_ |
| Grief  LIVIA 2.0  LIVIA 1 | 3.18(0.65)  3.14(0.64)  3.21(0.67) | 27  12  15 | 2.51(0.63)  2.59(0.54)  2.44(0.69) | 27  12  15 | 2.48(0.74)  2.61(0.63)  2.37(0.84) | 24  11  13 | *β*=-.77, *t*(25)=-4.63, *p*<.001, [-1.11; -.43], *d*=-1.19 | *β*=-.06, *t*(39.89)=-.26, *p*=.80, [-.57; .44] | *β*=.21, *t*(25)=.86, *p*=.40, [-.30; .73] | *β*=-.07, *t*(23.71)=-.39, *p*=.70, [-.44; .30], *d*=-.10 |
| Depression  LIVIA 2.0  LIVIA 1 | 0.86(0.45)  0.87(0.44)  0.86(0.48) | 27  12  15 | 0.71(0.46)  0.81(0.46)  0.63(0.45) | 27  12  15 | 0.54(0.43)  0.58(0.54)  0.51(0.33) | 22  10  12 | *β*=-.23, *t*(25)=-2.79, *p*=.01, [-.40; -.06], *d*=-.50 | *β*=.01, *t*(31.72)=.06, *p*=.95, [-.35; .37] | *β*=.16, *t*(25)=1.33, *p*=.19, [-.09; .42] | *β*=-.07, *t*(20.36)=-.81, *p*=.43, [-.25; .11], *d*=-.15 |
| Anxiety  LIVIA 2.0  LIVIA 1 | 0.89(0.59)  0.77(0.45)  0.98(0.69) | 27  12  15 | 0.69(0.58)  0.67(0.58)  0.71(0.61) | 27  12  15 | 0.58(0.55)  0.58(0.71)  0.57(0.41) | 22  10  12 | *β*=-.27, *t*(25)=-2.23, *p*=.03, [-.51; -.02], *d*=-.45 | *β*=-.21, *t*(33.58)=-.90, *p*=.38, [-.68; .26] | *β*=.16, *t*(25)=.89, *p*=.38, [-.21; .53] | *β*=-.03, *t*(20.73)=-.35, *p*=.73, [-.23; .17], *d*=-.05 |
| Well-being  LIVIA 2.0  LIVIA 1 | 5.09(0.95)  4.75(0.86)  5.36(0.96) | 27  12  15 | 5.22(0.87)  4.93(0.78)  5.45(0.89) | 27  12  15 | 5.31(1.06)  4.81(0.76)  5.72(1.12) | 22  10  12 | *β*=.09, *t*(25)=.52, *p*=.60, [-.27; .45], *d*=.10 | *β*=-.61, *t*(33.48)=-1.79, *p*=.08, [-1.3; .08] | *β*=.08, *t*(25)=.32, *p*=.75, [-.45; .63] | *β*=.21, *t*(20.98)=1.10, *p*=.28, [-.19; .60], *d*=.23 |
| Loneliness  LIVIA 2.0  LIVIA 1 | 2.19(0.56)  2.19(0.66)  2.19(0.96) | 27  12  15 | 2.06(0.50)  2.15(0.55)  1.99(0.47) | 27  12  15 | 2.09(0.58)  2.27(0.65)  1.94(0.49) | 22  10  12 | *β*=-.19, *t*(25)=-1.71, *p*=.10, [-.43; .04], *d*=-.36 | *β*=.01, *t*(34.35)=.02, *p*=.98, [-.42; .43] | *β*=.16, *t*(25)=.92, *p*=.37, [-.19; .50] | *β*=-.08, *t*(20.86)=-.94, *p*=.36, [-.24; .09], *d*=-.16 |
| Self-concept clarity  LIVIA 2.0  LIVIA 1 | 3.34(0.84)  3.47(1.02)  3.24(0.69) | 27  12  15 | 3.37(0.85)  3.45(0.81)  3.31(0.91) | 27  12  15 | 3.66(0.83)  3.73(0.71)  3.60(0.95) | 22  10  12 | *β*=.07, *t*(25)=.43, *p*=.67, [-.26; .39], *d*=.08 | *β*=.23, *t*(31.98)=.70, *p*=.49, [-.44; .91] | *β*=.09, *t*(25)=-.37, *p*=.71, [-.57; .40] | *β*=.26, *t*(20.94)=2.01, *p*=.06, [-.01; .53], *d*=.30 |
| Centrality of event  LIVIA 2.0  LIVIA 1 | 3.53(0.92)  3.43(0.79)  3.61(1.02) | 27  12  15 | 3.15(1.10)  3.16(0.98)  3.14(1.22) | 27  12  15 | 3.09(1.14)  3.39(1.16)  2.84(1.11) | 22  10  12 | *β*=-.47, *t*(25)=-2.15, *p*=.04, [-.91; -.02], *d*=-.45 | *β*=-.17, *t*(34.58)=-.44., *p*=.66, [-.98; .63] | *β*=.19, *t*(25)=.59, *p*=.56, [-.48; .86] | *β*=-.21, *t*(20.58)=-1.09, *p*=.29, [-.61; .19], *d*=-.19 |
| Self-continuity  LIVIA 2.0  LIVIA 1 | 2.72(0.93)  2.89(0.82)  2.58(1.01) | 27  12  15 | 2.68(0.98)  2.78(0.91)  2.60(1.05) | 27  12  15 | 2.98(1.07)  3.00(1.11)  2.97(1.08) | 22  10  12 | *β*=.02, *t*(25)=.13 *p*=.90, [-.33; .38], *d*=.02 | *β*=.31, *t*(31.81)=.83, *p*=.41, [-.45; 1.07] | *β*=-.13, *t*(25)=-.51, *p*=.61, [-.67; .40] | *β*=.39, *t*(21.08)=1.98, *p*=.06, [-.02; .79], *d*=.37 |

*Note*. Grief symptoms were assessed by using the Traumatic Grief Inventory; Depression symptoms were assessed by using the Patient Health Questionnaire (PHQ-9); Anxiety symptoms were assessed by using the General Anxiety Disorder-7 (GAD-7); Well-being was assessed by using the Flourishing Scale; Loneliness was assessed by using the UCLA Loneliness Scale; Identity scales used were the Self-Concept Clarity Scale, Centrality of Event Scale, and Self-Continuity items. ^a^ Estimates of fixed effects
