# Supplementary material for: A French-Language Web-Based Intervention Targeting Prolonged Grief Symptoms in People Who Are Bereaved and Separated: Randomized Controlled Trial
Source: JMIR Form Res. 2024 Oct 16;8:e57294. doi: 10.2196/57294 (PMC11525088; doi:10.2196/57294)
Supplement: Multimedia Appendix 3 [file formative_v8i1e57294_app3.pdf]

CONSORT-EHEALTH (V 1.6.1) - Submission/Publication Form

The CONSORT-EHEALTH checklist is intended for authors of randomized trials evaluating web-based and Internet-based applications/interventions, including mobile interventions, electronic games (incl multiplayer games), social media, certain telehealth applications, and other interactive and/or networked electronic applications. Some of the items (e.g. all subitems under item 5 - description of the intervention) may also be applicable for other study designs.

The goal of the CONSORT EHEALTH checklist and guideline is to be
a) a guide for reporting for authors of RCTs,
b) to form a basis for appraisal of an ehealth trial (in terms of validity)

CONSORT-EHEALTH items/subitems are MANDATORY reporting items for studies published in the Journal of Medical Internet Research and other journals / scientific societies endorsing the checklist.

Items numbered 1., 2., 3., 4a., 4b etc are original CONSORT or CONSORT-NPT (non-pharmacologic treatment) items.
Items with Roman numerals (i., ii, iii, iv etc.) are CONSORT-EHEALTH extensions/ clarifications.

As the CONSORT-EHEALTH checklist is still considered in a formative stage, we would ask that you also RATE ON A SCALE OF 1-5 how important/useful you feel each item is FOR THE PURPOSE OF THE CHECKLIST and reporting guideline (optional).

Mandatory reporting items are marked with a red *.
In the textboxes, either copy & paste the relevant sections from your manuscript into this form - please include any quotes from your manuscript in QUOTATION MARKS,
or answer directly by providing additional information not in the manuscript, or elaborating on why the item was not relevant for this study.

YOUR ANSWERS WILL BE PUBLISHED AS A SUPPLEMENTARY FILE TO YOUR PUBLICATION IN JMIR AND ARE CONSIDERED PART OF YOUR PUBLICATION (IF ACCEPTED).
Please fill in these questions diligently. Information will not be copyedited, so please use proper spelling and grammar, use correct capitalization, and avoid abbreviations.

DO NOT FORGET TO SAVE AS PDF _AND_ CLICK THE SUBMIT BUTTON SO YOUR ANSWERS ARE IN OUR DATABASE !!!

Citation Suggestion (if you append the pdf as Appendix we suggest to cite this paper in the caption):
Eysenbach G, CONSORT-EHEALTH Group
CONSORT-EHEALTH: Improving and Standardizing Evaluation Reports of Web-based and Mobile Health Interventions

J Med Internet Res 2011;13(4):e126
URL: http://www.jmir.org/2011/4/e126/
doi: 10.2196/jmir.1923
PMID: 22209829

anik.debrot@gmail.com Changer de compte

Non partagé

Brouillon enregistré

* Indique une question obligatoire

Your name *

First Last

Anik Debrot

Primary Affiliation (short), City, Country *

University of Toronto, Toronto, Canada

University of Lausanne, Lausanne, Switzerlan

Your e-mail address *

abc@gmail.com

anik.debrot@unil.ch

Title of your manuscript *

Provide the (draft) title of your manuscript.

A French-language Internet-based intervention targeting prolonged grief symptoms in bereaved and separated people: A randomized controlled trial

Name of your App/Software/Intervention *

If there is a short and a long/alternate name, write the short name first and add the long name in brackets.

LIVIA 2.0

Evaluated Version (if any)

e.g. "V1", "Release 2017-03-01", "Version 2.0.27913"

LIVIA 2.0

Language(s) *

What language is the intervention/app in? If multiple languages are available, separate by comma (e.g. "English, French")

French

URL of your Intervention Website or App

e.g. a direct link to the mobile app on app in appstore (itunes, Google Play), or URL of the website. If the intervention is a DVD or hardware, you can also link to an Amazon page.

Votre réponse

URL of an image/screenshot (optional)

Votre réponse

Accessibility *

Can an enduser access the intervention presently?

- ○ access is free and open
- ○ access only for special usergroups, not open
- ○ access is open to everyone, but requires payment/subscription/in-app purchases
- ◉ app/intervention no longer accessible
- ○ Autre :

Primary Medical Indication/Disease/Condition *

e.g. "Stress", "Diabetes", or define the target group in brackets after the condition, e.g. "Autism (Parents of children with)", "Alzheimers (Informal Caregivers of)"

Prolonged grief symptoms

Primary Outcomes measured in trial *

comma-separated list of primary outcomes reported in the trial

Prolonged grief symptoms, depressive sympt

Secondary/other outcomes

Are there any other outcomes the intervention is expected to affect?

Anxiety symptoms, feelings of loneliness, self-concept clarity, centrality of the loss, self-continuity

Recommended "Dose" *

What do the instructions for users say on how often the app should be used?

- ○ Approximately Daily
- ◉ Approximately Weekly
- ○ Approximately Monthly
- ○ Approximately Yearly
- ○ "as needed"
- ○ Autre :

Approx. Percentage of Users (starters) still using the app as recommended after 3 months *

- ○ unknown / not evaluated
- ○ 0-10%
- ○ 11-20%
- ○ 21-30%
- ○ 31-40%
- ○ 41-50%
- ◉ 51-60%
- ○ 61-70%
- ○ 71%-80%
- ○ 81-90%
- ○ 91-100%
- ○ Autre :

Overall, was the app/intervention effective? *

- ○ yes: all primary outcomes were significantly better in intervention group vs control
- ○ partly: SOME primary outcomes were significantly better in intervention group vs control
- ◉ no statistically significant difference between control and intervention
- ○ potentially harmful: control was significantly better than intervention in one or more outcomes
- ○ inconclusive: more research is needed
- ○ Autre :

Article Preparation Status/Stage *

At which stage in your article preparation are you currently (at the time you fill in this form)

- ○ not submitted yet - in early draft status
- ○ not submitted yet - in late draft status, just before submission
- ○ submitted to a journal but not reviewed yet
- ◉ submitted to a journal and after receiving initial reviewer comments
- ○ submitted to a journal and accepted, but not published yet
- ○ published
- ○ Autre :

Journal *

If you already know where you will submit this paper (or if it is already submitted), please provide the journal name (if it is not JMIR, provide the journal name under "other")

- ○ not submitted yet / unclear where I will submit this
- ○ Journal of Medical Internet Research (JMIR)
- ○ JMIR mHealth and UHealth
- ○ JMIR Serious Games
- ○ JMIR Mental Health
- ○ JMIR Public Health
- ◉ JMIR Formative Research
- ○ Other JMIR sister journal
- ○ Autre :

Is this a full powered effectiveness trial or a pilot/feasibility trial? *

- ○ Pilot/feasibility
- ◉ Fully powered

Manuscript tracking number *

If this is a JMIR submission, please provide the manuscript tracking number under "other" (The ms tracking number can be found in the submission acknowledgement email, or when you login as author in JMIR. If the paper is already published in JMIR, then the ms tracking number is the four-digit number at the end of the DOI, to be found at the bottom of each published article in JMIR)

- ○ no ms number (yet) / not (yet) submitted to / published in JMIR
- ◉ Autre : JFR ms#57294

TITLE AND ABSTRACT

1a) TITLE: Identification as a randomized trial in the title

1a) Does your paper address CONSORT item 1a? *

I.e does the title contain the phrase "Randomized Controlled Trial"? (if not, explain the reason under "other")

- (selected) yes
- Autre :

1a-i) Identify the mode of delivery in the title

Identify the mode of delivery. Preferably use “web-based” and/or “mobile” and/or “electronic game” in the title. Avoid ambiguous terms like “online”, “virtual”, “interactive”. Use “Internet-based” only if Intervention includes non-web-based Internet components (e.g. email), use “computer-based” or “electronic” only if offline products are used. Use “virtual” only in the context of “virtual reality” (3-D worlds). Use “online” only in the context of “online support groups”. Complement or substitute product names with broader terms for the class of products (such as “mobile” or “smart phone” instead of “iphone”), especially if the application runs on different platforms.

	1	2	3	4	5	
subitem not at all important	○	○	○	○	●	essential

Effacer la sélection

Does your paper address subitem 1a-i? *

Copy and paste relevant sections from manuscript title (include quotes in quotation marks "like this" to indicate direct quotes from your manuscript), or elaborate on this item by providing additional information not in the ms, or briefly explain why the item is not applicable/relevant for your study

"A French-language Web-based intervention"

1a-ii) Non-web-based components or important co-interventions in title

Mention non-web-based components or important co-interventions in title, if any (e.g., “with telephone support”).

	1	2	3	4	5	
subitem not at all important	(●)	( )	( )	( )	( )	essential

Effacer la sélection

Does your paper address subitem 1a-ii?

Copy and paste relevant sections from manuscript title (include quotes in quotation marks "like this" to indicate direct quotes from your manuscript), or elaborate on this item by providing additional information not in the ms, or briefly explain why the item is not applicable/relevant for your study

Votre réponse

1a-iii) Primary condition or target group in the title

Mention primary condition or target group in the title, if any (e.g., “for children with Type I Diabetes”) Example: A Web-based and Mobile Intervention with Telephone Support for Children with Type I Diabetes: Randomized Controlled Trial

	1	2	3	4	5	
subitem not at all important	( )	( )	( )	( )	(●)	essential

Effacer la sélection

Does your paper address subitem 1a-iii? *

Copy and paste relevant sections from manuscript title (include quotes in quotation marks "like this" to indicate direct quotes from your manuscript), or elaborate on this item by providing additional information not in the ms, or briefly explain why the item is not applicable/relevant for your study

"targeting prolonged grief symptoms in bereaved and separated people"

1b) ABSTRACT: Structured summary of trial design, methods, results, and conclusions

NPT extension: Description of experimental treatment, comparator, care providers, centers, and blinding status.

1b-i) Key features/functionalities/components of the intervention and comparator in the METHODS section of the ABSTRACT

Mention key features/functionalities/components of the intervention and comparator in the abstract. If possible, also mention theories and principles used for designing the site. Keep in mind the needs of systematic reviewers and indexers by including important synonyms. (Note: Only report in the abstract what the main paper is reporting. If this information is missing from the main body of text, consider adding it)

	1	2	3	4	5	
subitem not at all important	○	○	○	○	◉	essential

Effacer la sélection

Does your paper address subitem 1b-i? *

Copy and paste relevant sections from the manuscript abstract (include quotes in quotation marks "like this" to indicate direct quotes from your manuscript), or elaborate on this item by providing additional information not in the ms, or briefly explain why the item is not applicable/relevant for your study

LIVIA 2.0 was developed relying on theoretical and empirical findings on bereavement processes and IBIs, and is compared with LIVIA 1, which has already demonstrated its efficacy. We conducted a randomised controlled trial"

1b-ii) Level of human involvement in the METHODS section of the ABSTRACT

Clarify the level of human involvement in the abstract, e.g., use phrases like “fully automated” vs. “therapist/nurse/care provider/physician-assisted” (mention number and expertise of providers involved, if any). (Note: Only report in the abstract what the main paper is reporting. If this information is missing from the main body of text, consider adding it)

	1	2	3	4	5	
subitem not at all important	○	○	○	○	◉	essential

Effacer la sélection

Does your paper address subitem 1b-ii?

Copy and paste relevant sections from the manuscript abstract (include quotes in quotation marks "like this" to indicate direct quotes from your manuscript), or elaborate on this item by providing additional information not in the ms, or briefly explain why the item is not applicable/relevant for your study

"provided on-demand guidance to participants"

1b-iii) Open vs. closed, web-based (self-assessment) vs. face-to-face assessments in the METHODS section of the ABSTRACT

Mention how participants were recruited (online vs. offline), e.g., from an open access website or from a clinic or a closed online user group (closed usergroup trial), and clarify if this was a purely web-based trial, or there were face-to-face components (as part of the intervention or for assessment). Clearly say if outcomes were self-assessed through questionnaires (as common in web-based trials). Note: In traditional offline trials, an open trial (open-label trial) is a type of clinical trial in which both the researchers and participants know which treatment is being administered. To avoid confusion, use “blinded” or “unblinded” to indicated the level of blinding instead of “open”, as “open” in web-based trials usually refers to “open access” (i.e. participants can self-enrol). (Note: Only report in the abstract what the main paper is reporting. If this information is missing from the main body of text, consider adding it)

	1	2	3	4	5	
subitem not at all important	○	○	○	◉	○	essential

Effacer la sélection

Does your paper address subitem 1b-iii?

Copy and paste relevant sections from the manuscript abstract (include quotes in quotation marks "like this" to indicate direct quotes from your manuscript), or elaborate on this item by providing additional information not in the ms, or briefly explain why the item is not applicable/relevant for your study

"Outcomes were assessed exclusively through online questionnaires"

1b-iv) RESULTS section in abstract must contain use data

Report number of participants enrolled/assessed in each group, the use/uptake of the intervention (e.g., attrition/adherence metrics, use over time, number of logins etc.), in addition to primary/secondary outcomes. (Note: Only report in the abstract what the main paper is reporting. If this information is missing from the main body of text, consider adding it)

	1	2	3	4	5	
subitem not at all important	○	○	○	○	◉	essential

Effacer la sélection

Does your paper address subitem 1b-iv?

Copy and paste relevant sections from the manuscript abstract (include quotes in quotation marks "like this" to indicate direct quotes from your manuscript), or elaborate on this item by providing additional information not in the ms, or briefly explain why the item is not applicable/relevant for your study

"62 participants were randomized (Intent-To-Treat – ITT sample), 29 in LIVIA 2.0 and 33 in LIVIA 1 (control). The drop-out rate was 56.5%, leading to a final Per Protocol (PP) sample of 27 completers [...]. There were pre-post within group differences for both LIVIA programmes in the PP sample, with significant reductions in grief (d=-1.19), depressive (d=-.50), anxiety symptoms (d=-.45) and centrality of the loss (d=-.45). The same pattern was observed in the ITT sample. No difference was found in efficacy between the two

1b-v) CONCLUSIONS/DISCUSSION in abstract for negative trials

Conclusions/Discussions in abstract for negative trials: Discuss the primary outcome - if the trial is negative (primary outcome not changed), and the intervention was not used, discuss whether negative results are attributable to lack of uptake and discuss reasons. (Note: Only report in the abstract what the main paper is reporting. If this information is missing from the main body of text, consider adding it)

	1	2	3	4	5	
subitem not at all important	○	○	○	○	◉	essential

Effacer la sélection

Does your paper address subitem 1b-v?

Copy and paste relevant sections from the manuscript abstract (include quotes in quotation marks "like this" to indicate direct quotes from your manuscript), or elaborate on this item by providing additional information not in the ms, or briefly explain why the item is not applicable/relevant for your study

"The two grief-related IBIs were effective to diminish grief, depressive and anxiety symptoms for bereaved and separated participants. The analyses did not reveal any pre-post between-group differences, suggesting that the innovations brought to LIVIA 2.0 did not significantly affect the outcome compared to the original version. However, caution is warranted with the interpretation of the results given the limited power of the sample, which only allows the detection of medium effect sizes."

INTRODUCTION

2a) In INTRODUCTION: Scientific background and explanation of rationale

2a-i) Problem and the type of system/solution

Describe the problem and the type of system/solution that is object of the study: intended as stand-alone intervention vs. incorporated in broader health care program? Intended for a particular patient population? Goals of the intervention, e.g., being more cost-effective to other interventions, replace or complement other solutions? (Note: Details about the intervention are provided in “Methods” under 5)

	1	2	3	4	5	
subitem not at all important	○	○	○	○	◉	essential

Effacer la sélection

Does your paper address subitem 2a-i? *

Copy and paste relevant sections from the manuscript (include quotes in quotation marks "like this" to indicate direct quotes from your manuscript), or elaborate on this item by providing additional information not in the ms, or briefly explain why the item is not applicable/relevant for your study

"Distress following the loss of a loved one is a painful yet normal reaction. While most individuals recover over time, some experience prolonged grief symptoms, characterized by intense feelings of grief that persist for an extended period [1, 2]. Face-to-face interventions show moderate to large effect sizes to treat these symptoms [3, 4], but lack accessibility (e.g. [5, 6]. Web-based interventions (WBIs) can help improve accessibility and provide numerous efficient prevention and treatment programs for a variety of psychological difficulties [7, 8]. Notably, WBIs have demonstrated effectiveness in adressing prolonged grief symptoms, yielding moderate to large effect sizes [9, 10]. These interventions are generally based on methods derived from empirically supported face-to-face psychological interventions.
A common means to enhancing WBI effectiveness [11], including those targeting prolonged grief symptoms [9], is to provide guidance to participants (i.e., "any direct and bidirectional communication with the individual designed to support the clinical aspects of the intervention, facilitate intervention completion and/or achieve the desired clinical outcomes" [11], p. 230). However, recent evidence suggests that the impact of guidance on effect sizes is lower in more interactive internet interventions [13]. Additionally, when given the option, not all participants request guidance, yet the efficacy of a guidance on demand condition is similar to that of standard weekly guidance [14, 15]."

2a-ii) Scientific background, rationale: What is known about the (type of) system

Scientific background, rationale: What is known about the (type of) system that is the object of the study (be sure to discuss the use of similar systems for other conditions/ diagnoses, if appropiate), motivation for the study, i.e. what are the reasons for and what is the context for this specific study, from which stakeholder viewpoint is the study performed, potential impact of findings [2]. Briefly justify the choice of the comparator.

	1	2	3	4	5	
subitem not at all important	○	○	○	○	◉	essential

Effacer la sélection

Does your paper address subitem 2a-ii? *

Copy and paste relevant sections from the manuscript (include quotes in quotation marks "like this" to indicate direct quotes from your manuscript), or elaborate on this item by providing additional information not in the ms, or briefly explain why the item is not applicable/relevant for your study

"LIVIA 1 is an WBI program designed to treat prolonged grief symptoms following bereavement or separation [16]. Fundamental research indicates that both types of losses involve very similar underlying processes (e.g. [17, 18]). LIVIA 1 was assessed in German through a randomized controlled trial (RCT) [19] and in French through a noncontrolled trial [20]. These studies demonstrated that the same intervention can be efficiently administered to both populations. A detailed description of the LIVIA 1 intervention is available in the protocol by Brodbeck et al. [16]. For the present study, we developed LIVIA 2.0, an upgraded version of LIVIA 1. This program integrates recent developments in WBIs [21, 22] and incorporates various elements to enchance patient adherence and program efficacy whilst reducing the need for guidance. Specifically, a series of changes were designed to improve participant autonomy. First, we sent automated emails [23] in two situations: a) to announce to the participants that a new session is available and b) in case the participant has not accessed the intervention for seven consecutive days. Second, we more closely tailored the intervention to each participant in two ways: a) by providing automated individualized recommendations about the module completion order [24]; b) by proposing at each session a choice of different exercises that meet different situations or needs. More specifically, we evaluated at the first session each participant's priorities and recommended the order of the modules accordingly. In each session, we provided three choices of exercises so that the participant could chose what suited their needs best. Third, we evaluated, promoted and encouraged the use of personal resources based on a validated self-assessement tool, the AERES [22]. Finally, relying on research showing the benefits of augmented interactivity [21, 25, 26], we developed more interactive content in the form of psychoeducation videos and quizzes. Apart from the introductory and concluding sessions, the structure of LIVIA 2.0 revolves around four modules focusing on key cognitive-behavioural therapy topics: thoughts, behaviours and emotions. Moreover, we developed a module based on empirical cognitive psychopathological knowledge that addresses identity and memory processes, which are crucial for adapting to loss [27, 28]. Autobiographical memory refers to memories from past personal experiences. It serves to maintain self-continuity and provides the ability to stay oriented in the world and pursue goals [29, 30]. In the grief context, the loss of a significant other is often a life-changing event that can disrupt one's life story, sense of self, and future plans [28]. Addressing these disturbances can therefore play a crucial role in alleviating prolonged grief symptoms by helping individuals develop a more adaptive and coherent sense of self. Given these considerations, we aimed to include measures of three key identity-related variables in our study: a) self-continuity, which refers to the perception of a coherent connection between one's past, present, and future self [31]; b) self-concept clarity, which refers to the clear and coherent understanding of one's own traits, beliefs, and values [32]; and c) event centrality, which refers to the extent to which individuals construct the traumatic event as a reference point to understand themselves and the world [33]. By doing so, we aim to provide a more comprehensive understanding of how the LIVIA interventions impact these facets of identity.

The innovations in LIVIA 2.0 were also developed on the theoretical and empirical literature on grief and romantic dissolution. Theoretically, we relied on one of the most influential models of coping with loss, the Dual Process Model (DPM) of Coping with Bereavement [34, 35]. According to this model, instead of progressing through consecutive phases, individuals oscillate between focusing on the loss and focusing on restoration from the loss. This oscillation is considered a natural and necessary process for coping with loss. Additionally, evidence suggests that DPM-based interventions may be more effective than traditional ones [36]. LIVIA 2.0 was designed to mimic the oscillation process by alternating between loss- and restoration-focused sessions within each of its four modules. Furthermore, LIVIA 2.0 incorporates recent empirical findings related to loss

four modules. Furthermore, LIVIA 2.0 incorporates recent empirical findings related to loss into its content and exercises, such as self-compassion exercises, which predict better grief recovery [37, 38]. Exploratory analyses of the utilization and potential impact of the innovations included in LIVIA 2.0 were conducted, in particular in relation to guidance requirements, automated e-mails, reliance on personal resources, and the identity module [39]. Given the combination of empirically-based changes implemented in LIVIA 2.0, we

2b) In INTRODUCTION: Specific objectives or hypotheses

Does your paper address CONSORT subitem 2b? *

Copy and paste relevant sections from the manuscript (include quotes in quotation marks "like this" to indicate direct quotes from your manuscript), or elaborate on this item by providing additional information not in the ms, or briefly explain why the item is not applicable/relevant for your study

"Our main hypotheses are as follows: (1) both LIVIA 1 and LIVIA 2.0 will increase participants' well-being and decrease their mental health symptoms at post-test and follow-up; (2) LIVIA 2.0 will be more efficient than LIVIA 1 across all outcomes; and (3) LIVIA 2.0 will have a lower dropout rate than LIVIA 1. Additionally, we will compare participant satisfaction between both versions."

METHODS

3a) Description of trial design (such as parallel, factorial) including allocation ratio

Does your paper address CONSORT subitem 3a? *

Copy and paste relevant sections from the manuscript (include quotes in quotation marks "like this" to indicate direct quotes from your manuscript), or elaborate on this item by providing additional information not in the ms, or briefly explain why the item is not applicable/relevant for your study

"This study is a monocentric, single-blinded, 2-arm RCT comparing the efficacy of two versions of a French-language WBI –LIVIA 1 and LIVIA 2.0 – designed to alleviate mental health symptoms and enhance the well-being of individuals experiencing prolonged grief symptoms following the loss of a loved one."

3b) Important changes to methods after trial commencement (such as eligibility criteria), with reasons

Does your paper address CONSORT subitem 3b? *

Copy and paste relevant sections from the manuscript (include quotes in quotation marks "like this" to indicate direct quotes from your manuscript), or elaborate on this item by providing additional information not in the ms, or briefly explain why the item is not applicable/relevant for your study

We changed some of the aims due to the much smaller sample size than anticipated. "First, the comparison of guidance requirements between LIVIA 2.0 and LIVIA 1, as well as part of the qualitative investigation of the semantic content of the responses to the LIVIA 2.0 exercises, are discussed in other publications [39, 41]. Second, the smaller sample size obtained, compared to the target, does not provide sufficient statistical power to analyse the short-term effectiveness of each LIVIA 2.0 module on participants' weekly moods, feelings of loneliness, and prolonged grief symptoms, nor to explore the role of multiple measures as moderators of the program's efficacy. "

Accordingly, the statistical analyses were also changed:
"Due to difficulties in participant recruitment, our sample size was significantly smaller than targeted. Post-hoc analysis revealed that the statistical power of the PP sample was adequate (.80) to compute within-between person interactions for a medium effect size, but insufficient to detect small within-between effect sizes (.18) or medium between-person differences (.33). Consequently, we adapted the analyses and decided not to conduct most secondary analyses."

3b-i) Bug fixes, Downtimes, Content Changes

Bug fixes, Downtimes, Content Changes: ehealth systems are often dynamic systems. A description of changes to methods therefore also includes important changes made on the intervention or comparator during the trial (e.g., major bug fixes or changes in the functionality or content) (5-iii) and other “unexpected events” that may have influenced study design such as staff changes, system failures/downtimes, etc. [2].

	1	2	3	4	5	
subitem not at all important	(●)	( )	( )	( )	( )	essential

Effacer la sélection

Does your paper address subitem 3b-i?

Copy and paste relevant sections from the manuscript (include quotes in quotation marks "like this" to indicate direct quotes from your manuscript), or elaborate on this item by providing additional information not in the ms, or briefly explain why the item is not applicable/relevant for your study

Fortunately, we did not experienced bugs or downtimes during the study, as we had carefully tested the intervention previously.

4a) Eligibility criteria for participants

Does your paper address CONSORT subitem 4a? *

Copy and paste relevant sections from the manuscript (include quotes in quotation marks "like this" to indicate direct quotes from your manuscript), or elaborate on this item by providing additional information not in the ms, or briefly explain why the item is not applicable/relevant for your study

"Inclusion criteria were: a) having experienced bereavement or separation more than six months prior to participation, b) feeling the need for support to cope with the loss, c) being over 18 years old, d) having regular access to the internet and some computer/Internet literacy, e) speaking French fluently, and f) having provided written approval of the informed consent form.
Exclusion criteria were: a) the presence of moderate to acute suicidality (assessed before the start of the programme), b) the presence of severe psychological or somatic disorders requiring immediate treatment, c) concomitant psychotherapy, d) the prescription or dosage change of psychoactive drugs in the month prior to or during the programme, e) the inability to follow the study procedures, and f) enrolment of the investigators, their family members, employees, and other dependent people."

4a-i) Computer / Internet literacy

Computer / Internet literacy is often an implicit “de facto” eligibility criterion - this should be explicitly clarified.

	1	2	3	4	5	
subitem not at all important	○	○	○	◉	○	essential

Effacer la sélection

Does your paper address subitem 4a-i?

Copy and paste relevant sections from the manuscript (include quotes in quotation marks "like this" to indicate direct quotes from your manuscript), or elaborate on this item by providing additional information not in the ms, or briefly explain why the item is not applicable/relevant for your study

Please see above: we mention this criteria in the manuscript.

4a-ii) Open vs. closed, web-based vs. face-to-face assessments:

Open vs. closed, web-based vs. face-to-face assessments: Mention how participants were recruited (online vs. offline), e.g., from an open access website or from a clinic, and clarify if this was a purely web-based trial, or there were face-to-face components (as part of the intervention or for assessment), i.e., to what degree got the study team to know the participant. In online-only trials, clarify if participants were quasi-anonymous and whether having multiple identities was possible or whether technical or logistical measures (e.g., cookies, email confirmation, phone calls) were used to detect/prevent these.

	1	2	3	4	5	
subitem not at all important	○	○	○	◉	○	essential

Effacer la sélection

Does your paper address subitem 4a-ii? *

Copy and paste relevant sections from the manuscript (include quotes in quotation marks "like this" to indicate direct quotes from your manuscript), or elaborate on this item by providing additional information not in the ms, or briefly explain why the item is not applicable/relevant for your study

"Participants were recruited from French-speaking regions of Switzerland. Recruitment was conducted by contacting associations (e.g., grief- and divorce-related organizations, senior citizens groups, and neighbourhood associations), engaging with media outlets (radio, television, newspapers), distributing flyers in public locations (e.g., beauty salons, churches), emailing university student groups, promoting the study through social media (Facebook, Instagram), and posting advertisements on research facility websites. Recruitment lasted from May 2022 to January 2023."

4a-iii) Information giving during recruitment

Information given during recruitment. Specify how participants were briefed for recruitment and in the informed consent procedures (e.g., publish the informed consent documentation as appendix, see also item X26), as this information may have an effect on user self-selection, user expectation and may also bias results.

	1	2	3	4	5	
subitem not at all important	○	○	○	◉	○	essential

Effacer la sélection

Does your paper address subitem 4a-iii?

Copy and paste relevant sections from the manuscript (include quotes in quotation marks "like this" to indicate direct quotes from your manuscript), or elaborate on this item by providing additional information not in the ms, or briefly explain why the item is not applicable/relevant for your study

"All participants filled in an informed consent form that they downloaded online, together with an information sheet. We provided our contact information on several places for them to contact us in case of questions. To conform with the Swiss law and ethical practices, participants had to sign the informed consent by hand. They then could either scan and e-mail us the informed consent, or send it by mail. "

4b) Settings and locations where the data were collected

Does your paper address CONSORT subitem 4b? *

Copy and paste relevant sections from the manuscript (include quotes in quotation marks "like this" to indicate direct quotes from your manuscript), or elaborate on this item by providing additional information not in the ms, or briefly explain why the item is not applicable/relevant for your study

"All outcomes were assessed via self-reported questionnaires that were completed online by participants."

4b-i) Report if outcomes were (self-)assessed through online questionnaires

Clearly report if outcomes were (self-)assessed through online questionnaires (as common in web-based trials) or otherwise.

	1	2	3	4	5	
subitem not at all important	○	○	○	○	●	essential

Effacer la sélection

Does your paper address subitem 4b-i? *

Copy and paste relevant sections from the manuscript (include quotes in quotation marks "like this" to indicate direct quotes from your manuscript), or elaborate on this item by providing additional information not in the ms, or briefly explain why the item is not applicable/relevant for your study

"All outcomes were assessed via self-reported questionnaires that were completed online by participants."

4b-ii) Report how institutional affiliations are displayed

Report how institutional affiliations are displayed to potential participants [on ehealth media], as affiliations with prestigious hospitals or universities may affect volunteer rates, use, and reactions with regards to an intervention.(Not a required item – describe only if this may bias results)

	1	2	3	4	5	
subitem not at all important	○	○	●	○	○	essential

Effacer la sélection

Does your paper address subitem 4b-ii?

Copy and paste relevant sections from the manuscript (include quotes in quotation marks "like this" to indicate direct quotes from your manuscript), or elaborate on this item by providing additional information not in the ms, or briefly explain why the item is not applicable/relevant for your study

"Our institutional affiliation was displayed on all recruitment material (posters, website, social media posts, flyers). Moreover, the affiliation was also always mentioned on the media mentioning our project (radio and press articles)."

5) The interventions for each group with sufficient details to allow replication, including how and when they were actually administered

5-i) Mention names, credential, affiliations of the developers, sponsors, and owners

Mention names, credential, affiliations of the developers, sponsors, and owners [6] (if authors/evaluators are owners or developer of the software, this needs to be declared in a “Conflict of interest” section or mentioned elsewhere in the manuscript).

	1	2	3	4	5	
subitem not at all important	○	○	○	◉	○	essential

Effacer la sélection

Does your paper address subitem 5-i?

Copy and paste relevant sections from the manuscript (include quotes in quotation marks "like this" to indicate direct quotes from your manuscript), or elaborate on this item by providing additional information not in the ms, or briefly explain why the item is not applicable/relevant for your study

"LIVIA 1 is a 10-session self-help intervention developed to address prolonged grief symptoms resulting from the death of or the separation/divorce from a romantic partner developped by Brodbeck and colleagues [16]. [...] LIVIA 2.0 is a psychological WBI developped by the authors of the study. [...] Both interventions were hosted on a website developed by RationalK." There is no other sponsor than the university and the funder (SNSF, mentionned in the manuscript). There is no conflict of interest to declare.

5-ii) Describe the history/development process

Describe the history/development process of the application and previous formative evaluations (e.g., focus groups, usability testing), as these will have an impact on adoption/use rates and help with interpreting results.

	1	2	3	4	5	
subitem not at all important	○	○	○	◉	○	essential

Effacer la sélection

Does your paper address subitem 5-ii?

Copy and paste relevant sections from the manuscript (include quotes in quotation marks "like this" to indicate direct quotes from your manuscript), or elaborate on this item by providing additional information not in the ms, or briefly explain why the item is not applicable/relevant for your study

"Previous versions of the modules were qualitatively pretested on small samples within the frame of Master theses [43–48]. The intervention was adapted according to the

5-iii) Revisions and updating

Revisions and updating. Clearly mention the date and/or version number of the application/intervention (and comparator, if applicable) evaluated, or describe whether the intervention underwent major changes during the evaluation process, or whether the development and/or content was “frozen” during the trial. Describe dynamic components such as news feeds or changing content which may have an impact on the replicability of the intervention (for unexpected events see item 3b).

	1	2	3	4	5	
subitem not at all important	○	○	○	○	◉	essential

Effacer la sélection

Does your paper address subitem 5-iii?

Copy and paste relevant sections from the manuscript (include quotes in quotation marks "like this" to indicate direct quotes from your manuscript), or elaborate on this item by providing additional information not in the ms, or briefly explain why the item is not applicable/relevant for your study

"The content of the intervention was frozen during the present trial."

5-iv) Quality assurance methods

Provide information on quality assurance methods to ensure accuracy and quality of information provided [1], if applicable.

	1	2	3	4	5	
subitem not at all important	○	○	○	●	○	essential

Effacer la sélection

Does your paper address subitem 5-iv?

Copy and paste relevant sections from the manuscript (include quotes in quotation marks "like this" to indicate direct quotes from your manuscript), or elaborate on this item by providing additional information not in the ms, or briefly explain why the item is not applicable/relevant for your study

A formal quality check was not performed. However, the content of the intervention was first based on a validated and expert-developed intervention, and second, was further developed by knowledgeable researchers and based on empirical knowledge.

5-v) Ensure replicability by publishing the source code, and/or providing screenshots/screen-capture video, and/or providing flowcharts of the algorithms used

Ensure replicability by publishing the source code, and/or providing screenshots/screen-capture video, and/or providing flowcharts of the algorithms used. Replicability (i.e., other researchers should in principle be able to replicate the study) is a hallmark of scientific reporting.

	1	2	3	4	5	
subitem not at all important	○	○	●	○	○	essential

Effacer la sélection

Does your paper address subitem 5-v?

Copy and paste relevant sections from the manuscript (include quotes in quotation marks "like this" to indicate direct quotes from your manuscript), or elaborate on this item by providing additional information not in the ms, or briefly explain why the item is not applicable/relevant for your study

We have not provided source codes, visual documentation of the intervention, and algorithm flowcharts. The source codes for data analysis are available upon request.

5-vi) Digital preservation

Digital preservation: Provide the URL of the application, but as the intervention is likely to change or disappear over the course of the years; also make sure the intervention is archived (Internet Archive, webcitation.org, and/or publishing the source code or screenshots/videos alongside the article). As pages behind login screens cannot be archived, consider creating demo pages which are accessible without login.

	1	2	3	4	5	
subitem not at all important	○	○	●	○	○	essential

Effacer la sélection

Does your paper address subitem 5-vi?

Copy and paste relevant sections from the manuscript (include quotes in quotation marks "like this" to indicate direct quotes from your manuscript), or elaborate on this item by providing additional information not in the ms, or briefly explain why the item is not applicable/relevant for your study

This item was not addressed. Examples from the intervention platform are still available from the developers.

5-vii) Access

Access: Describe how participants accessed the application, in what setting/context, if they had to pay (or were paid) or not, whether they had to be a member of specific group. If known, describe how participants obtained “access to the platform and Internet” [1]. To ensure access for editors/reviewers/readers, consider to provide a “backdoor” login account or demo mode for reviewers/readers to explore the application (also important for archiving purposes, see vi).

	1	2	3	4	5	
subitem not at all important	○	○	○	○	◉	essential

Effacer la sélection

Does your paper address subitem 5-vii? *

Copy and paste relevant sections from the manuscript (include quotes in quotation marks "like this" to indicate direct quotes from your manuscript), or elaborate on this item by providing additional information not in the ms, or briefly explain why the item is not applicable/relevant for your study

"Participants who were randomized received a link to create an account on the intervention platform corresponding to their intervention. They could then freely access the intervention at the rythme they wished, but a weekly session was recommended."

5-viii) Mode of delivery, features/functionalities/components of the intervention and comparator, and the theoretical framework

Describe mode of delivery, features/functionalities/components of the intervention and comparator, and the theoretical framework [6] used to design them (instructional strategy [1], behaviour change techniques, persuasive features, etc., see e.g., [7, 8] for terminology). This includes an in-depth description of the content (including where it is coming from and who developed it) [1]," whether [and how] it is tailored to individual circumstances and allows users to track their progress and receive feedback" [6]. This also includes a description of communication delivery channels and – if computer-mediated communication is a component – whether communication was synchronous or asynchronous [6]. It also includes information on presentation strategies [1], including page design principles, average amount of text on pages, presence of hyperlinks to other resources, etc. [1].

	1	2	3	4	5	
subitem not at all important	○	○	○	○	◉	essential

Effacer la sélection

Does your paper address subitem 5-viii? *

Copy and paste relevant sections from the manuscript (include quotes in quotation marks "like this" to indicate direct quotes from your manuscript), or elaborate on this item by providing additional information not in the ms, or briefly explain why the item is not applicable/relevant for your study

"LIVIA 1 is a 10-session self-help intervention developed to address prolonged grief symptoms resulting from the death of or the separation/divorce from a romantic partner developed by Brodbeck and colleagues (for more information, see [16]). Participants are encouraged to complete one session per week, and working through exercises provided in downloadable PDF files. Each session takes approximately 60 minutes to complete, and participants must follow the prescribed order of sessions. It includes various texts, audio files, exercises, and interactive quizzes, and serves as the control condition [40]. The efficacy of LIVIA 1 has been previously demonstrated [19].
LIVIA 2.0 is a psychological WBI developed by the authors of the study consisting of 10 sessions [40, 42], each taking 30-45 minutes to complete. It includes an introductory session, eight sessions divided into four modules, and a concluding session. The modules cover four main themes: cognitions, emotions, behaviours, and identity. Based on the results of a short questionnaire, an individual recommendation of the order in which to complete the modules is provided at the end of the introductory session. Theoretically anchored in the Dual Process Model [35], each module comprises a first session focused on loss and a second on restoration. Each session contains psychoeducational information and three versions of an exercise related to the session’s main theme. Participants are expected to complete at least one exercise per session; they can chose from three options the exercice that best suits them, but can complete all the exercises if they wish to. LIVIA 2.0 incorporates various exercises, texts, audio and video files, and interactive quizzes. Participant in this condition can access a maximum of one session per week and receive an automated e-mail when a new session is available. In this study, it serves as the experimental condition. Previous versions of the modules were qualitatively pretested on small samples within the frame of Master theses [43–48]. The intervention was adapted according to the results. The content of the intervention was frozen during the present trial. Both interventions were hosted on a website developed by RationalK SàRL. "

5-ix) Describe use parameters

Describe use parameters (e.g., intended “doses” and optimal timing for use). Clarify what instructions or recommendations were given to the user, e.g., regarding timing, frequency, heaviness of use, if any, or was the intervention used ad libitum.

	1	2	3	4	5	
subitem not at all important	○	○	○	◉	○	essential

Effacer la sélection

Does your paper address subitem 5-ix?

Copy and paste relevant sections from the manuscript (include quotes in quotation marks "like this" to indicate direct quotes from your manuscript), or elaborate on this item by providing additional information not in the ms, or briefly explain why the item is not applicable/relevant for your study

"Participants [in LIVIA 1] are encouraged to complete one session per week for estimated duration of about one hour."
"[...]10 sessions [40, 42], each taking 30-45 minutes to complete. [...] Participant in this condition [LIVIA 2.0] can access a maximum of one session per week"

5-x) Clarify the level of human involvement

Clarify the level of human involvement (care providers or health professionals, also technical assistance) in the e-intervention or as co-intervention (detail number and expertise of professionals involved, if any, as well as “type of assistance offered, the timing and frequency of the support, how it is initiated, and the medium by which the assistance is delivered”. It may be necessary to distinguish between the level of human involvement required for the trial, and the level of human involvement required for a routine application outside of a RCT setting (discuss under item 21 – generalizability).

	1	2	3	4	5	
subitem not at all important	○	○	○	○	◉	essential

Effacer la sélection

Does your paper address subitem 5-x?

Copy and paste relevant sections from the manuscript (include quotes in quotation marks "like this" to indicate direct quotes from your manuscript), or elaborate on this item by providing additional information not in the ms, or briefly explain why the item is not applicable/relevant for your study

"In both study conditions, participants received automated e-mails when they had not accessed the intervention platform for a week and could ask for guidance on demand."

5-xi) Report any prompts/reminders used

Report any prompts/reminders used: Clarify if there were prompts (letters, emails, phone calls, SMS) to use the application, what triggered them, frequency etc. It may be necessary to distinguish between the level of prompts/reminders required for the trial, and the level of prompts/reminders for a routine application outside of a RCT setting (discuss under item 21 – generalizability).

	1	2	3	4	5	
subitem not at all important	○	○	○	○	◉	essential

Effacer la sélection

Does your paper address subitem 5-xi? *

Copy and paste relevant sections from the manuscript (include quotes in quotation marks "like this" to indicate direct quotes from your manuscript), or elaborate on this item by providing additional information not in the ms, or briefly explain why the item is not applicable/relevant for your study

"In both study conditions, participants received automated e-mails when they had not accessed the intervention platform for a week"
"Participant in this condition [LIVIA 2.0] can access a maximum of one session per week and receive an automated e-mail when a new session is available.

5-xii) Describe any co-interventions (incl. training/support)

Describe any co-interventions (incl. training/support): Clearly state any interventions that are provided in addition to the targeted eHealth intervention, as ehealth intervention may not be designed as stand-alone intervention. This includes training sessions and support [1]. It may be necessary to distinguish between the level of training required for the trial, and the level of training for a routine application outside of a RCT setting (discuss under item 21 – generalizability.

	1	2	3	4	5	
subitem not at all important	(selected)	○	○	○	○	essential

Effacer la sélection

Does your paper address subitem 5-xii? *

Copy and paste relevant sections from the manuscript (include quotes in quotation marks "like this" to indicate direct quotes from your manuscript), or elaborate on this item by providing additional information not in the ms, or briefly explain why the item is not applicable/relevant for your study

No training was required and, besides the guidance and automated e-mails mentioned above, no additional support was provided to participants.

6a) Completely defined pre-specified primary and secondary outcome measures, including how and when they were assessed

Does your paper address CONSORT subitem 6a? *

Copy and paste relevant sections from the manuscript (include quotes in quotation marks "like this" to indicate direct quotes from your manuscript), or elaborate on this item by providing additional information not in the ms, or briefly explain why the item is not applicable/relevant for your study

"Primary outcome measures
All outcomes were assessed via self-reported questionnaires that were completed online by participants. Prolonged grief symptoms were assessed with the Traumatic Grief Inventory Self-Report [49][50]. This 18-item self-report measure assesses the presence of symptoms on a 5-point scale, ranging from 1 (never) to 5 (always) [49]. This inventory is designed to evaluate symptoms of persistent complex bereavement disorder, as defined in the Diagnostic and Statistical Manual of Mental Disorders (5th edition [1]), and prolonged grief disorder, as per the International Classification of Diseases (11th edition [51]). It demonstrates good reliability and validity in identifying individuals at risk for prolonged grief disorder.
Depression symptoms were assessed with the Patient Health Questionnaire-9 [52], a 9-item measure of depression with adequate reliability and validity [53]. This questionnaire assesses various depressive symptoms over the previous two weeks on a scale ranging from 0 (never) to 3 (almost every day).
Well-being was measured with the French version [54] of the Flourishing Scale [55], a brief 8-item instrument of self-perceived success in important life areas such as relationships, self-esteem, purpose, and optimism. This scale assesses eudemonic well-being, a broader conception of conventional well-being measures. Participants responded to items such as “I lead a purposeful and meaningful life” on a scale ranging from 1 (strongly disagree) to 7 (strongly agree).
Secondary outcome measures
Anxiety symptoms were measured with the Generalized Anxiety Scale [56, 57] , which includes 7 items (e.g., “Feeling nervous, anxious, or on edge”). Participants rated the frequency of symptoms over the previous two weeks on a 4-point Likert scale (0 = not at all; 3 = nearly every day).
Feelings of loneliness were assessed with the University of California Los Angeles Loneliness Scale [58, 59] which contains 10 positive items (e.g., “I feel in tune with the people around me”) and 10 negative items (e.g., “I lack companionship”). Participants responded on a 4-point scale (1 = never to 4 = often).
Identity-related concepts were evaluated with three different scales. First, the 12-item Self-Concept Clarity Scale in its French version [32, 60] assesses the clarity, consistency, and stability of self-beliefs. Participants answered on a 5-point scale ranging from 1 (strongly disagree) to 5 (strongly agree). Second, the Centrality of Event Scale [33], French version by Ceschi [61], assesses the extent to which a distressing life event serves as a reference point for personal identity and meaning attribution to other experiences. Responses were rated on a 5-point scale (1 = totally disagree to 5 = totally agree). Finally, three items assessed self-continuity [62]: “I am the same person as I always was,” “With time a lot of things have changed, but I’m still the same person,” and “I am a different person than I was in the past.” These items were evaluated on a 5-point scale (ranging from 1 = does not apply to me at all to 5 = fully applies to me)."

6a-i) Online questionnaires: describe if they were validated for online use and apply CHERRIES items to describe how the questionnaires were designed/ deployed

If outcomes were obtained through online questionnaires, describe if they were validated for online use and apply CHERRIES items to describe how the questionnaires were designed/deployed [9].

	1	2	3	4	5	
subitem not at all important	○	◉	○	○	○	essential

Effacer la sélection

Does your paper address subitem 6a-i?

Copy and paste relevant sections from manuscript text

"All outcomes were assessed via self-reported questionnaires that were completed online by participants on the REDCAP platform [49, 50] of the CHUV (Centre Hospitalier Universitaire Vaudois). Participants were invited to complete the different questionnaires via an e-mail containing an individual link. Up to three reminders were sent at each stage (pre- and postest and follow-up) if the questionnaires were not answered within a week. "

6a-ii) Describe whether and how “use” (including intensity of use/dosage) was defined/measured/monitored

Describe whether and how “use” (including intensity of use/dosage) was defined/ measured/monitored (logins, logfile analysis, etc.). Use/adoption metrics are important process outcomes that should be reported in any ehealth trial.

	1	2	3	4	5	
subitem not at all important	○	○	○	◉	○	essential

Effacer la sélection

Does your paper address subitem 6a-ii?

Copy and paste relevant sections from manuscript text

We provide the percentage of randomized having completed the posttest assessment and the percentage of people included the in per-protocol analyses.

6a-iii) Describe whether, how, and when qualitative feedback from participants was obtained

Describe whether, how, and when qualitative feedback from participants was obtained (e.g., through emails, feedback forms, interviews, focus groups).

	1	2	3	4	5	
subitem not at all important	○	○	○	●	○	essential

Effacer la sélection

Does your paper address subitem 6a-iii?

Copy and paste relevant sections from manuscript text

"[...] satisfaction with the programme was measured with a translated and adapted version of the Client Satisfaction Questionnaire adapted to Internet-based interventions (CSQ-I [65]). We included open-ended questions to obtain qualitative feedback on the intervention."

Besides, a qualitative study whereby we interviewed by phone participants who had droped out of the study was conducted. It was decided posthoc to conduct the study, given the

6b) Any changes to trial outcomes after the trial commenced, with reasons

Does your paper address CONSORT subitem 6b? *

Copy and paste relevant sections from the manuscript (include quotes in quotation marks "like this" to indicate direct quotes from your manuscript), or elaborate on this item by providing additional information not in the ms, or briefly explain why the item is not applicable/relevant for your study

No change was made on the outcomes after the trial commenced.

7a) How sample size was determined

NPT: When applicable, details of whether and how the clustering by care provides or centers was addressed

7a-i) Describe whether and how expected attrition was taken into account when calculating the sample size

Describe whether and how expected attrition was taken into account when calculating the sample size.

	1	2	3	4	5	
subitem not at all important	○	○	○	◉	○	essential

Effacer la sélection

Does your paper address subitem 7a-i?

Copy and paste relevant sections from manuscript title (include quotes in quotation marks "like this" to indicate direct quotes from your manuscript), or elaborate on this item by providing additional information not in the ms, or briefly explain why the item is not applicable/relevant for your study

"We conducted a power analysis with G*Power (Version 3.1.9.2 [43]) for ANOVA of repeated measures involving within-between interactions that would enable us to compare the efficacy of the 2 interventions. Based on a probability level of 0.05 and a power of 0.8, to detect a small effect size of f =0.1 (as we expect small differences between the 2 programs), a sample size of 164 participants is needed. Expecting a dropout rate of 30% at the 3-month follow-up, we aim to have 234 participants at posttest."

Note that this information was provided in the protocol article (A. Debrot, M. Kheyar, L. Efinger, L. Berthoud, and V. Pomini, "Supporting people who have lost a close person by bereavement or separation: Protocol of a randomized controlled trial comparing two French-language Internet-based interventions," JMIR Research Protocols, vol. 11, no. 6, e39026, 2022, doi: 10.2196/39026.)

7b) When applicable, explanation of any interim analyses and stopping guidelines

Does your paper address CONSORT subitem 7b? *

Copy and paste relevant sections from the manuscript (include quotes in quotation marks "like this" to indicate direct quotes from your manuscript), or elaborate on this item by providing additional information not in the ms, or briefly explain why the item is not applicable/relevant for your study

We did not planned interim analyses. We planned to stop the recruitment when the target sample size was obtained or when the project was finished.

8a) Method used to generate the random allocation sequence

NPT: When applicable, how care providers were allocated to each trial group

Does your paper address CONSORT subitem 8a? *

Copy and paste relevant sections from the manuscript (include quotes in quotation marks "like this" to indicate direct quotes from your manuscript), or elaborate on this item by providing additional information not in the ms, or briefly explain why the item is not applicable/relevant for your study

"We used the Randomization module in REDCap (Harris et al., 2009; Harris et al., 2019), which generates the randomization automatically. "

8b) Type of randomisation; details of any restriction (such as blocking and block size)

Does your paper address CONSORT subitem 8b? *

Copy and paste relevant sections from the manuscript (include quotes in quotation marks "like this" to indicate direct quotes from your manuscript), or elaborate on this item by providing additional information not in the ms, or briefly explain why the item is not applicable/relevant for your study

"[we used] randomization blocks of ten persons with an allocation of 1:1."

9) Mechanism used to implement the random allocation sequence (such as sequentially numbered containers), describing any steps taken to conceal the sequence until interventions were assigned

Does your paper address CONSORT subitem 9? *

Copy and paste relevant sections from the manuscript (include quotes in quotation marks "like this" to indicate direct quotes from your manuscript), or elaborate on this item by providing additional information not in the ms, or briefly explain why the item is not applicable/relevant for your study

"We applied a single-blinded randomization strategy stratified according to the gender and loss type (bereavement vs. separation)"

10) Who generated the random allocation sequence, who enrolled participants, and who assigned participants to interventions

Does your paper address CONSORT subitem 10? *

Copy and paste relevant sections from the manuscript (include quotes in quotation marks "like this" to indicate direct quotes from your manuscript), or elaborate on this item by providing additional information not in the ms, or briefly explain why the item is not applicable/relevant for your study

"We used the Randomization module in REDCap (Harris et al., 2009; Harris et al., 2019), which generates the randomization automatically. "

11a) If done, who was blinded after assignment to interventions (for example, participants, care providers, those assessing outcomes) and how

NPT: Whether or not administering co-interventions were blinded to group assignment

11a-i) Specify who was blinded, and who wasn't

Specify who was blinded, and who wasn't. Usually, in web-based trials it is not possible to blind the participants [1, 3] (this should be clearly acknowledged), but it may be possible to blind outcome assessors, those doing data analysis or those administering co-interventions (if any).

	1	2	3	4	5	
subitem not at all important	○	○	○	◉	○	essential

Effacer la sélection

Does your paper address subitem 11a-i? *

Copy and paste relevant sections from the manuscript (include quotes in quotation marks "like this" to indicate direct quotes from your manuscript), or elaborate on this item by providing additional information not in the ms, or briefly explain why the item is not applicable/relevant for your study

"We applied a single-blinded randomization strategy "

Note that we did not reveal the version of the intervention, nor provided information in the recruitment process that could allow participants to guess in which condition they were.

11a-ii) Discuss e.g., whether participants knew which intervention was the "intervention of interest" and which one was the "comparator"

Informed consent procedures (4a-ii) can create biases and certain expectations - discuss e.g., whether participants knew which intervention was the "intervention of interest" and which one was the "comparator".

	1	2	3	4	5	
subitem not at all important	○	○	○	○	◉	essential

Effacer la sélection

Does your paper address subitem 11a-ii?

Copy and paste relevant sections from the manuscript (include quotes in quotation marks "like this" to indicate direct quotes from your manuscript), or elaborate on this item by providing additional information not in the ms, or briefly explain why the item is not applicable/relevant for your study

"We applied a single-blinded randomization strategy "

Note that we did not reveal the version of the intervention, nor provided information in the recruitment process that could allow participants to guess in which condition they were.

11b) If relevant, description of the similarity of interventions

(this item is usually not relevant for ehealth trials as it refers to similarity of a placebo or sham intervention to a active medication/intervention)

Does your paper address CONSORT subitem 11b? *

Copy and paste relevant sections from the manuscript (include quotes in quotation marks "like this" to indicate direct quotes from your manuscript), or elaborate on this item by providing additional information not in the ms, or briefly explain why the item is not applicable/relevant for your study

Indeed, this items is not relevant for our study.

12a) Statistical methods used to compare groups for primary and secondary outcomes

NPT: When applicable, details of whether and how the clustering by care providers or centers was addressed

Does your paper address CONSORT subitem 12a? *

Copy and paste relevant sections from the manuscript (include quotes in quotation marks "like this" to indicate direct quotes from your manuscript), or elaborate on this item by providing additional information not in the ms, or briefly explain why the item is not applicable/relevant for your study

"To test hypotheses 1) and 2), we employed multilevel mixed-effects models with repeated measures data to evaluate the efficacy of LIVIA 2.0 compared to LIVIA 1 and the stability of the effects. These models account for the dependency of the data and the correlation of repeated measures within individuals [67]."

12a-i) Imputation techniques to deal with attrition / missing values

Imputation techniques to deal with attrition / missing values: Not all participants will use the intervention/comparator as intended and attrition is typically high in ehealth trials. Specify how participants who did not use the application or dropped out from the trial were treated in the statistical analysis (a complete case analysis is strongly discouraged, and simple imputation techniques such as LOCF may also be problematic [4]).

	1	2	3	4	5	
subitem not at all important	○	○	○	◉	○	essential

Effacer la sélection

Does your paper address subitem 12a-i? *

Copy and paste relevant sections from the manuscript (include quotes in quotation marks "like this" to indicate direct quotes from your manuscript), or elaborate on this item by providing additional information not in the ms, or briefly explain why the item is not applicable/relevant for your study

"[multilevel mixed-effects models with repeated measures data utilise] all available data from each participant and estimating parameters for missing values."

12b) Methods for additional analyses, such as subgroup analyses and adjusted analyses

Does your paper address CONSORT subitem 12b? *

Copy and paste relevant sections from the manuscript (include quotes in quotation marks "like this" to indicate direct quotes from your manuscript), or elaborate on this item by providing additional information not in the ms, or briefly explain why the item is not applicable/relevant for your study

"For the descriptive characteristics of the sample at baseline, we tested differences between both experimental arms using t-tests for continuous variables and chi-square tests for categorical variables, based on the ITT sample. "

X26) REB/IRB Approval and Ethical Considerations [recommended as subheading under "Methods"] (not a CONSORT item)

X26-i) Comment on ethics committee approval

	1	2	3	4	5	
subitem not at all important	○	○	○	◉	○	essential

Effacer la sélection

Does your paper address subitem X26-i?

Copy and paste relevant sections from the manuscript (include quotes in quotation marks "like this" to indicate direct quotes from your manuscript), or elaborate on this item by providing additional information not in the ms, or briefly explain why the item is not applicable/relevant for your study

"The research protocol was approved by a federally-recognised state ethics committee (Commission cantonale d'éthique de la recherche sur l'être humain, CER-VD, BASEC reference number: 2021-D0086) and the Swiss Agency for Therapeutic Products (Swissmedic; reference number: 102667545) in accordance with Swiss Ordinance 810.306 on Clinical Trials with Medical Devices. The trial was registered in the ClinicalTrials.gov database (reference number: NCT05219760)."

x26-ii) Outline informed consent procedures

Outline informed consent procedures e.g., if consent was obtained offline or online (how? Checkbox, etc.?), and what information was provided (see 4a-ii). See [6] for some items to be included in informed consent documents.

	1	2	3	4	5	
subitem not at all important	○	○	○	◉	○	essential

Effacer la sélection

Does your paper address subitem X26-ii?

Copy and paste relevant sections from the manuscript (include quotes in quotation marks "like this" to indicate direct quotes from your manuscript), or elaborate on this item by providing additional information not in the ms, or briefly explain why the item is not applicable/relevant for your study

"All participants filled in an informed consent form that they downloaded online, together with an information sheet. We provided our contact information on several places for them to contact us in case of questions. To conform with the Swiss law and ethical practices, participants had to sign the informed consent by hand. They then could either scan and e-mail us the informed consent, or send it by mail."

X26-iii) Safety and security procedures

Safety and security procedures, incl. privacy considerations, and any steps taken to reduce the likelihood or detection of harm (e.g., education and training, availability of a hotline)

	1	2	3	4	5	
subitem not at all important	○	○	○	○	◉	essential

Effacer la sélection

Does your paper address subitem X26-iii?

Copy and paste relevant sections from the manuscript (include quotes in quotation marks "like this" to indicate direct quotes from your manuscript), or elaborate on this item by providing additional information not in the ms, or briefly explain why the item is not applicable/relevant for your study

We paid ample attention to safety issues in our study. A detailed account of all safety procedures can be found in the protocol approved by the local ethical committee.

RESULTS

13a) For each group, the numbers of participants who were randomly assigned, received intended treatment, and were analysed for the primary outcome

NPT: The number of care providers or centers performing the intervention in each group and the number of patients treated by each care provider in each center

Does your paper address CONSORT subitem 13a? *

Copy and paste relevant sections from the manuscript (include quotes in quotation marks "like this" to indicate direct quotes from your manuscript), or elaborate on this item by providing additional information not in the ms, or briefly explain why the item is not applicable/relevant for your study

"27 participants were finally included into the per protocol (PP) analyses (LIVIA 1: n=15; LIVIA 2: n=12)"

13b) For each group, losses and exclusions after randomisation, together with reasons

Does your paper address CONSORT subitem 13b? (NOTE: Preferably, this is shown in a CONSORT flow diagram) *

Copy and paste relevant sections from the manuscript (include quotes in quotation marks "like this" to indicate direct quotes from your manuscript), or elaborate on this item by providing additional information not in the ms, or briefly explain why the item is not applicable/relevant for your study

"Due to drop-outs and exclusions for not meeting inclusion criteria (starting another treatment during the programme (LIVIA 2.0: n=3; LIVIA 1: n=2), not completing at least one full session(LIVIA 2.0: n=3; LIVIA 1: n=2)), 27 participants were finally included into the per protocol (PP) analyses (LIVIA 1: n=15; LIVIA 2: n=12)."

13b-i) Attrition diagram

Strongly recommended: An attrition diagram (e.g., proportion of participants still logging in or using the intervention/comparator in each group plotted over time, similar to a survival curve) or other figures or tables demonstrating usage/dose/engagement.

	1	2	3	4	5	
subitem not at all important	○	○	○	◉	○	essential

Effacer la sélection

Does your paper address subitem 13b-i?

Copy and paste relevant sections from the manuscript or cite the figure number if applicable (include quotes in quotation marks "like this" to indicate direct quotes from your manuscript), or elaborate on this item by providing additional information not in the ms, or briefly explain why the item is not applicable/relevant for your study

We have largely addressed attrition and user rates, but we have not depicted this in a diagram.

14a) Dates defining the periods of recruitment and follow-up

Does your paper address CONSORT subitem 14a? *

Copy and paste relevant sections from the manuscript (include quotes in quotation marks "like this" to indicate direct quotes from your manuscript), or elaborate on this item by providing additional information not in the ms, or briefly explain why the item is not applicable/relevant for your study

"Recruitment lasted from May 2022 to January 2023. The last participant terminated the follow-up in August 2023. "

14a-i) Indicate if critical “secular events” fell into the study period

Indicate if critical “secular events” fell into the study period, e.g., significant changes in Internet resources available or “changes in computer hardware or Internet delivery resources”

	1	2	3	4	5	
subitem not at all important	○	○	○	◉	○	essential

Effacer la sélection

Does your paper address subitem 14a-i?

Copy and paste relevant sections from the manuscript (include quotes in quotation marks "like this" to indicate direct quotes from your manuscript), or elaborate on this item by providing additional information not in the ms, or briefly explain why the item is not applicable/relevant for your study

No such event occurred during the study period.

14b) Why the trial ended or was stopped (early)

Does your paper address CONSORT subitem 14b? *

Copy and paste relevant sections from the manuscript (include quotes in quotation marks "like this" to indicate direct quotes from your manuscript), or elaborate on this item by providing additional information not in the ms, or briefly explain why the item is not applicable/relevant for your study

"We concluded recruitment due to time and funding constraints."

15) A table showing baseline demographic and clinical characteristics for each group

NPT: When applicable, a description of care providers (case volume, qualification, expertise, etc.) and centers (volume) in each group

Does your paper address CONSORT subitem 15? *

Copy and paste relevant sections from the manuscript (include quotes in quotation marks "like this" to indicate direct quotes from your manuscript), or elaborate on this item by providing additional information not in the ms, or briefly explain why the item is not applicable/relevant for your study

This information can be found in Tables 2 and 3.

15-i) Report demographics associated with digital divide issues

In ehealth trials it is particularly important to report demographics associated with digital divide issues, such as age, education, gender, social-economic status, computer/Internet/ehealth literacy of the participants, if known.

	1	2	3	4	5	
subitem not at all important	○	○	○	◉	○	essential

Effacer la sélection

Does your paper address subitem 15-i? *

Copy and paste relevant sections from the manuscript (include quotes in quotation marks "like this" to indicate direct quotes from your manuscript), or elaborate on this item by providing additional information not in the ms, or briefly explain why the item is not applicable/relevant for your study

This information can be found in Table 2. Computer/Internet/ehealth literacy was not assessed.

16) For each group, number of participants (denominator) included in each analysis and whether the analysis was by original assigned groups

16-i) Report multiple “denominators” and provide definitions

Report multiple “denominators” and provide definitions: Report N’s (and effect sizes) “across a range of study participation [and use] thresholds” [1], e.g., N exposed, N consented, N used more than x times, N used more than y weeks, N participants “used” the intervention/comparator at specific pre-defined time points of interest (in absolute and relative numbers per group). Always clearly define “use” of the intervention.

	1	2	3	4	5	
subitem not at all important	○	○	○	○	◉	essential

Effacer la sélection

Does your paper address subitem 16-i? *

Copy and paste relevant sections from the manuscript (include quotes in quotation marks "like this" to indicate direct quotes from your manuscript), or elaborate on this item by providing additional information not in the ms, or briefly explain why the item is not applicable/relevant for your study

We report whether the analyses were performed on the intention to treat or per protocol sample. We report the denominator if it deviates from the size of the sample.

16-ii) Primary analysis should be intent-to-treat

Primary analysis should be intent-to-treat, secondary analyses could include comparing only “users”, with the appropriate caveats that this is no longer a randomized sample (see 18-i).

	1	2	3	4	5	
subitem not at all important	○	○	○	◉	○	essential

Effacer la sélection

Does your paper address subitem 16-ii?

Copy and paste relevant sections from the manuscript (include quotes in quotation marks "like this" to indicate direct quotes from your manuscript), or elaborate on this item by providing additional information not in the ms, or briefly explain why the item is not applicable/relevant for your study

We report intention to treat analyses in the main manuscript and per protocol analyses (with truly "users") in the supplement and comment in the manuscript on the (small) found differences.

17a) For each primary and secondary outcome, results for each group, and the estimated effect size and its precision (such as 95% confidence interval)

Does your paper address CONSORT subitem 17a? *

Copy and paste relevant sections from the manuscript (include quotes in quotation marks "like this" to indicate direct quotes from your manuscript), or elaborate on this item by providing additional information not in the ms, or briefly explain why the item is not applicable/relevant for your study

We provide this information in Table 4.

17a-i) Presentation of process outcomes such as metrics of use and intensity of use

In addition to primary/secondary (clinical) outcomes, the presentation of process outcomes such as metrics of use and intensity of use (dose, exposure) and their operational definitions is critical. This does not only refer to metrics of attrition (13-b) (often a binary variable), but also to more continuous exposure metrics such as “average session length”. These must be accompanied by a technical description how a metric like a “session” is defined (e.g., timeout after idle time) [1] (report under item 6a).

	1	2	3	4	5	
subitem not at all important	○	○	○	◉	○	essential

Effacer la sélection

Does your paper address subitem 17a-i?

Copy and paste relevant sections from the manuscript (include quotes in quotation marks "like this" to indicate direct quotes from your manuscript), or elaborate on this item by providing additional information not in the ms, or briefly explain why the item is not applicable/relevant for your study

Yes, apart from the primary/secondary outcomes, we have mentioned drop out and completion rates.

17b) For binary outcomes, presentation of both absolute and relative effect sizes is recommended

Does your paper address CONSORT subitem 17b? *

Copy and paste relevant sections from the manuscript (include quotes in quotation marks "like this" to indicate direct quotes from your manuscript), or elaborate on this item by providing additional information not in the ms, or briefly explain why the item is not applicable/relevant for your study

This does not apply to our data.

18) Results of any other analyses performed, including subgroup analyses and adjusted analyses, distinguishing pre-specified from exploratory

Does your paper address CONSORT subitem 18? *

Copy and paste relevant sections from the manuscript (include quotes in quotation marks "like this" to indicate direct quotes from your manuscript), or elaborate on this item by providing additional information not in the ms, or briefly explain why the item is not applicable/relevant for your study

We present Per Protocol analyses in the Supplementary file.

18-i) Subgroup analysis of comparing only users

A subgroup analysis of comparing only users is not uncommon in ehealth trials, but if done, it must be stressed that this is a self-selected sample and no longer an unbiased sample from a randomized trial (see 16-iii).

	1	2	3	4	5	
subitem not at all important	○	○	◉	○	○	essential

Effacer la sélection

Does your paper address subitem 18-i?

Copy and paste relevant sections from the manuscript (include quotes in quotation marks "like this" to indicate direct quotes from your manuscript), or elaborate on this item by providing additional information not in the ms, or briefly explain why the item is not applicable/relevant for your study

Given the small sample size, we did not performed subgroup analyses.

19) All important harms or unintended effects in each group
(for specific guidance see CONSORT for harms)

Does your paper address CONSORT subitem 19? *

Copy and paste relevant sections from the manuscript (include quotes in quotation marks "like this" to indicate direct quotes from your manuscript), or elaborate on this item by providing additional information not in the ms, or briefly explain why the item is not applicable/relevant for your study

This does not apply to our study as no adverse advents occurred.

19-i) Include privacy breaches, technical problems

Include privacy breaches, technical problems. This does not only include physical “harm” to participants, but also incidents such as perceived or real privacy breaches [1], technical problems, and other unexpected/unintended incidents. “Unintended effects” also includes unintended positive effects [2].

	1	2	3	4	5	
subitem not at all important	○	○	○	◉	○	essential

Effacer la sélection

Does your paper address subitem 19-i?

Copy and paste relevant sections from the manuscript (include quotes in quotation marks "like this" to indicate direct quotes from your manuscript), or elaborate on this item by providing additional information not in the ms, or briefly explain why the item is not applicable/relevant for your study

A few, not critical technical problems occurred. Participants could contact us via e-mail or via the contact form of the intervention website. All problems were resolved within max. 3 days.

19-ii) Include qualitative feedback from participants or observations from staff/ researchers

Include qualitative feedback from participants or observations from staff/researchers, if available, on strengths and shortcomings of the application, especially if they point to unintended/unexpected effects or uses. This includes (if available) reasons for why people did or did not use the application as intended by the developers.

	1	2	3	4	5	
subitem not at all important	○	○	○	◉	○	essential

Effacer la sélection

Does your paper address subitem 19-ii?

Copy and paste relevant sections from the manuscript (include quotes in quotation marks "like this" to indicate direct quotes from your manuscript), or elaborate on this item by providing additional information not in the ms, or briefly explain why the item is not applicable/relevant for your study

Due to the already important size of the manuscript, we addressed this issues in another paper.

Efinger, L., Kheyar, M., Pomini, V., Berthoud, L., Wicki, R., & Debrot, A. (2024). Innovations to enhance engagement and efficacy in a mHealth psychological intervention targeting grief: Lessons learned and future directions. In T. F. Heston (Ed.), Biomedical Engineering. A comprehensive overview of telemedicine. IntechOpen.

DISCUSSION

22) Interpretation consistent with results, balancing benefits and harms, and considering other relevant evidence

NPT: In addition, take into account the choice of the comparator, lack of or partial blinding, and unequal expertise of care providers or centers in each group

22-i) Restate study questions and summarize the answers suggested by the data, starting with primary outcomes and process outcomes (use)

Restate study questions and summarize the answers suggested by the data, starting with primary outcomes and process outcomes (use).

	1	2	3	4	5	
subitem not at all important	○	○	○	○	◉	essential

Effacer la sélection

Does your paper address subitem 22-i? *

Copy and paste relevant sections from the manuscript (include quotes in quotation marks "like this" to indicate direct quotes from your manuscript), or elaborate on this item by providing additional information not in the ms, or briefly explain why the item is not applicable/relevant for your study

"This study aimed to compared two Web-Based Interventions (WBIs) for individuals with prolonged grief symptoms due to either death or romantic separation: LIVIA 1, an established program serving as the control condition, and LIVIA 2.0, a newly developed programme. The present study demonstrated that both programmes were effective in reducing grief, depression symptoms as well as the centrality of the loss and to improve self-concept clarity. However, no effect was found for other outcomes. Moreover, no difference emerged between both programmes' efficacy, drop-out rates and satisfaction level."

22-ii) Highlight unanswered new questions, suggest future research

Highlight unanswered new questions, suggest future research.

	1	2	3	4	5	
subitem not at all important	○	○	○	◉	○	essential

Effacer la sélection

Does your paper address subitem 22-ii?

Copy and paste relevant sections from the manuscript (include quotes in quotation marks "like this" to indicate direct quotes from your manuscript), or elaborate on this item by providing additional information not in the ms, or briefly explain why the item is not applicable/relevant for your study

We make several suggestions for future research throughout the discussion.

"Given these observations, future research should explore the potential mediating role of decreased event centrality on grief symptoms using larger samples [28, 73]. "
"However, these findings need replication given the small sample size. "
"Future research should further explore the factors contributing to participant' satisfaction and retention, such as those proposed by Ritterband et al. [79] for behaviour change by WBIs."
"Tailoring interventions to the specific needs of sub-samples might be beneficial. Developing a more sophisticated specification algorithm could improve content adjustment according to participant profiles [83], potentially incorporating a therapeutic chatbot and AI technologies [84]."
"In response to the high dropout rate, we are conducting interviews with participants who discontinued the programme to understand their motives and derive strategies for better retention. Additionally, a detailed analysis of browsing behaviours would enable to understand how the programme is used. This would provide reliable objective secondary data, allowing for example for improvements in (differential) indication. To obtain more conclusive results, it is essential to gather a larger sample by recruiting participants from other French-speaking European countries. While technological advancements facilitate this process, heterogeneous regulations can complicate it [85]. Finally, regarding a better tailoring of the intervention, adopting a co-design approach [86] could be beneficial. This could involve organizing focus groups with the target population and conducting interviews with key stakeholders, such as grief therapists. "

20) Trial limitations, addressing sources of potential bias, imprecision, and, if relevant, multiplicity of analyses

20-i) Typical limitations in ehealth trials

Typical limitations in ehealth trials: Participants in ehealth trials are rarely blinded. Ehealth trials often look at a multiplicity of outcomes, increasing risk for a Type I error. Discuss biases due to non-use of the intervention/usability issues, biases through informed consent procedures, unexpected events.

	1	2	3	4	5	
subitem not at all important	○	○	○	○	◉	essential

Effacer la sélection

Does your paper address subitem 20-i? *

Copy and paste relevant sections from the manuscript (include quotes in quotation marks "like this" to indicate direct quotes from your manuscript), or elaborate on this item by providing additional information not in the ms, or briefly explain why the item is not applicable/relevant for your study

On top of the specific limitations of our trial, we discuss typical limitations in ehealth trials.
"As often in e-health trials, the present study examined a multiplicity of outcomes, increasing the risk of Type I error [85]. Moreover, in the present analyses, due to the limited sample size, we did not examined the influence of the extent to which participants used the intervention. Finally, the rather complex informed consent process requiring a hand-written signature might have discouraged some people from participation and thus biased

21) Generalisability (external validity, applicability) of the trial findings

NPT: External validity of the trial findings according to the intervention, comparators, patients, and care providers or centers involved in the trial

21-i) Generalizability to other populations

Generalizability to other populations: In particular, discuss generalizability to a general Internet population, outside of a RCT setting, and general patient population, including applicability of the study results for other organizations

	1	2	3	4	5	
subitem not at all important	○	○	○	○	◉	essential

Effacer la sélection

Does your paper address subitem 21-i?

Copy and paste relevant sections from the manuscript (include quotes in quotation marks "like this" to indicate direct quotes from your manuscript), or elaborate on this item by providing additional information not in the ms, or briefly explain why the item is not applicable/relevant for your study

"The sample, although presented a large diversity in terms of age, gender, type of loss and employment type, thus well representing the population of people with grief symptoms. However, there was an overrepresentation of people with an University degree. "

21-ii) Discuss if there were elements in the RCT that would be different in a routine application setting

Discuss if there were elements in the RCT that would be different in a routine application setting (e.g., prompts/reminders, more human involvement, training sessions or other co-interventions) and what impact the omission of these elements could have on use, adoption, or outcomes if the intervention is applied outside of a RCT setting.

	1	2	3	4	5	
subitem not at all important	○	○	○	◉	○	essential

Effacer la sélection

Does your paper address subitem 21-ii?

Copy and paste relevant sections from the manuscript (include quotes in quotation marks "like this" to indicate direct quotes from your manuscript), or elaborate on this item by providing additional information not in the ms, or briefly explain why the item is not applicable/relevant for your study

"The results and observations from the present study might provide valuable insights for clinical application. First, given the high attrition rate during the recruitment process (only 27% of interested individuals were randomized), it is likely that recruitment could improve in a more naturalistic setting. Second, offering the programme in a blended therapy setting, as commonly practiced in clinical environments [8], would allow customisation of the timing of the content proposed by the LIVIA programmes."

OTHER INFORMATION

23) Registration number and name of trial registry

Does your paper address CONSORT subitem 23? *

Copy and paste relevant sections from the manuscript (include quotes in quotation marks "like this" to indicate direct quotes from your manuscript), or elaborate on this item by providing additional information not in the ms, or briefly explain why the item is not applicable/relevant for your study

"The trial was registered in the ClinicalTrials.gov database (reference number: NCT05219760)."

24) Where the full trial protocol can be accessed, if available

Does your paper address CONSORT subitem 24? *

Cite a Multimedia Appendix, other reference, or copy and paste relevant sections from the manuscript (include quotes in quotation marks "like this" to indicate direct quotes from your manuscript), or elaborate on this item by providing additional information not in the ms, or briefly explain why the item is not applicable/relevant for your study

Yes, we refer to the protocol paper in the methods section and the full proposal for the ethic committee can be found on ClinicalTrials.gov with the reference number (direct link: https://clinicaltrials.gov/study/NCT05219760?intr=livia&rank=1)

25) Sources of funding and other support (such as supply of drugs), role of funders

Does your paper address CONSORT subitem 25? *

Copy and paste relevant sections from the manuscript (include quotes in quotation marks "like this" to indicate direct quotes from your manuscript), or elaborate on this item by providing additional information not in the ms, or briefly explain why the item is not applicable/relevant for your study

"This study was supported by the SNSF grant 100014_182840 awarded to Anik Debrot and Valentino Pomini."

X27) Conflicts of Interest (not a CONSORT item)

X27-i) State the relation of the study team towards the system being evaluated

In addition to the usual declaration of interests (financial or otherwise), also state the relation of the study team towards the system being evaluated, i.e., state if the authors/evaluators are distinct from or identical with the developers/sponsors of the intervention.

	1	2	3	4	5	
subitem not at all important	○	○	○	○	◉	essential

Effacer la sélection

Does your paper address subitem X27-i?

Copy and paste relevant sections from the manuscript (include quotes in quotation marks "like this" to indicate direct quotes from your manuscript), or elaborate on this item by providing additional information not in the ms, or briefly explain why the item is not applicable/relevant for your study

We state in the Method section that we, the authors, developed the content of the tested intervention (LIVIA 2.0).

"LIVIA 2.0 is a psychological WBI developed by the authors of the study consisting of 10 sessions [40, 42], each taking 30-45 minutes to complete. "

About the CONSORT EHEALTH checklist

As a result of using this checklist, did you make changes in your manuscript? *

- ○ yes, major changes
- ◉ yes, minor changes
- ○ no

What were the most important changes you made as a result of using this checklist?

We report the ITT analyses in the manuscript and the PP analyses in the Supplementary file. We refer to Web-based interventions (WBIs) rather than Internet-based intervention (IBIs). We provide more details about the outcomes assessment, the procedure and the interventions.

How much time did you spend on going through the checklist INCLUDING making changes in your manuscript *

About 3 full days

Your answer must have a minimum of 25 characters.

As a result of using this checklist, do you think your manuscript has improved? *

- (●) yes
- ( ) no
- ( ) Autre :

Would you like to become involved in the CONSORT EHEALTH group?

This would involve for example becoming involved in participating in a workshop and writing an "Explanation and Elaboration" document

- (●) yes
- ( ) no
- ( ) Autre :

Effacer la sélection

Any other comments or questions on CONSORT EHEALTH

Votre réponse

STOP - Save this form as PDF before you click submit

To generate a record that you filled in this form, we recommend to generate a PDF of this page (on a Mac, simply select "print" and then select "print as PDF") before you submit it.

When you submit your (revised) paper to JMIR, please upload the PDF as supplementary file.

Don't worry if some text in the textboxes is cut off, as we still have the complete information in our database. Thank you!

Final step: Click submit !

Click submit so we have your answers in our database!

Envoyer

Effacer le formulaire

N'envoyez jamais de mots de passe via Google Forms.

Ce contenu n'est ni rédigé, ni cautionné par Google. Signaler un cas d'utilisation abusive - Conditions d'utilisation - Règles de confidentialité

Google Forms
